# Bactericidal effectors of the *Stenotrophomonas maltophilia* type IV secretion system: functional definition of the nuclease TfdA and structural determination of TfcB

**DOI:** 10.1128/mbio.01198-24

**Published:** 2024-06-04

**Authors:** Brandi L. Cobe, Supratim Dey, George Minasov, Nicole Inniss, Karla J. F. Satchell, Nicholas P. Cianciotto

**Affiliations:** 1 Department of Microbiology-Immunology, Northwestern University Feinberg School of Medicine, Chicago, Illinois, USA; 2 Center for Structural Biology of Infectious Diseases, Northwestern University Feinberg School of Medicine, Chicago, Illinois, USA; Institut Pasteur, Paris, France

**Keywords:** *Stenotrophomonas maltophilia*, T4SS, antibacterial effectors, interbacterial competition, bactericidal activity, GHH nuclease, DNase, chitinase, peptidoglycan hydrolase

## Abstract

*Stenotrophomonas maltophilia* expresses a type IV protein secretion system (T4SS) that promotes contact-dependent killing of other bacteria and does so partly by secreting the effector TfcB. Here, we report the structure of TfcB, comprising an N-terminal domain similar to the catalytic domain of glycosyl hydrolase (GH-19) chitinases and a C-terminal domain for recognition and translocation by the T4SS. Utilizing a two-hybrid assay to measure effector interactions with the T4SS coupling protein VirD4, we documented the existence of five more T4SS substrates. One of these was protein 20845, an annotated nuclease. A *S. maltophilia* mutant lacking the gene for 20845 was impaired for killing *Escherichia coli*, *Klebsiella pneumoniae*, and *Pseudomonas aeruginosa*. Moreover, the cloned 20845 gene conferred robust toxicity, with the recombinant *E. coli* being rescued when 20845 was co-expressed with its cognate immunity protein. The 20845 effector was an 899 amino-acid protein, comprised of a GHH-nuclease domain in its N-terminus, a large central region of indeterminant function, and a C-terminus for secretion. Engineered variants of the 20845 gene that had mutations in the predicted catalytic site did not impede *E. coli*, indicating that the antibacterial effect of 20845 involves its nuclease activity. Using flow cytometry with DNA staining, we determined that 20845, but not its mutant variants, confers a loss in DNA content of target bacteria. Database searches revealed that uncharacterized homologs of 20845 occur within a range of bacteria. These data indicate that the *S. maltophilia* T4SS promotes interbacterial competition through the action of multiple toxic effectors, including a potent, novel DNase.

**IMPORTANCE:**

*Stenotrophomonas maltophilia* is a multi-drug-resistant, Gram-negative bacterium that is an emerging pathogen of humans. Patients with cystic fibrosis are particularly susceptible to *S. maltophilia* infection. In hospital water systems and various types of infections, *S. maltophilia* co-exists with other bacteria, including other pathogens such as *Pseudomonas aeruginosa*. We previously demonstrated that *S. maltophilia* has a functional VirB/D4 type VI protein secretion system (T4SS) that promotes contact-dependent killing of other bacteria. Since most work on antibacterial systems involves the type VI secretion system, this observation remains noteworthy. Moreover, *S. maltophilia* currently stands alone as a model for a human pathogen expressing an antibacterial T4SS. Using biochemical, genetic, and cell biological approaches, we now report both the discovery of a novel antibacterial nuclease (TfdA) and the first structural determination of a bactericidal T4SS effector (TfcB).

## INTRODUCTION

The genus *Stenotrophomonas* includes at least 22 species of Gram-negative bacteria that are found in water, soil, and plant material (https://lpsn.dsmz.de/genus/stenotrophomonas) (1–4). Notable among the *Stenotrophomonas* species is *Stenotrophomonas maltophilia*, an opportunistic pathogen that causes, among other things, pneumonia and bacteremia (5–7). *S. maltophilia* is problematic in patients with cystic fibrosis (CF), where it increases the chance of lung exacerbations (8–10). *S. maltophilia* has also significantly infected coronavirus disease of 2019 (COVID-19) patients (11, 12). *S. maltophilia* infections are often difficult to treat due to the multi-drug-resistant nature of associated strains (13–15). To survive in its various niches, *S. maltophilia* utilizes biofilm formation and a range of surface and secreted factors, including flagella, siderophore, and enzymes secreted by a type II secretion system (16–26).

Recently, we determined that most isolates of *S. maltophilia* also encode a VirB/VirD4 type IV protein secretion system (T4SS) (27). Based on the characterization of a *virB10* mutant of the clinical isolate K279a, the *S. maltophilia* T4SS promotes apoptosis of macrophages while impeding the death of lung epithelial cells (27). Interestingly, the T4SS also has antibacterial activity, reducing, in a contact-dependent manner, the colony forming units (CFUs) of clinical and environmental isolates of *Pseudomonas aeruginosa*, *Pseudomonas mendocina*, and *Escherichia coli* (*Ec*) (27, 28). This antibacterial activity was confirmed by others, for example, in short-term co-incubations, *S. maltophilia* K279a lyses strains of *P. aeruginosa* and *E. coli* (29). Given that *S. maltophilia* and *P. aeruginosa* often co-exist in hospital water systems and various types of infections, especially those in the lungs (30–42), further knowledge of the antibacterial effects of the *S. maltophilia* T4SS was relevant for understanding human infection. We posited that the antimicrobial effect of the *S. maltophilia* T4SS is due to the delivery of toxic proteins (effectors) into target bacteria. A bioinformatic screen identified 13 potential effectors, most of which were conserved among clinical and environmental strains of *S. maltophilia* (27, 28). Mutants of *S. maltophilia* K279a lacking the putative effectors RS14245 (alternate locus tag: smlt2990) or RS14255 (smlt2992) were impaired for killing *E. coli* and clinical isolates of *P. aeruginosa*, including the ones obtained from the lungs of patients with CF (28). Compatible with this, protein similarity searches using Basic Local Alignment Search Tool Protein (BLASTP) and structural prediction software suggested that the ~46 kDa RS14245 is a lipase and the ~37 kDa RS14255 is a chitinase-like enzyme that might hydrolyze peptidoglycan (28). RS14245 and RS14255 were named TfcA and TfcB for type four secreted bactericidal effectors A and B, respectively (28).

In contrast to the large amount of data on bacterial T4SSs affecting higher-order hosts (43–48) and the many studies on the antibacterial effect of type VI secretion systems (T6SS), type VII secretion systems (T7SS), and contact-dependent inhibition systems (49–54), knowledge of the antibacterial role of T4SSs is very limited. Indeed, prior to the initial analyses of the *S. maltophilia* T4SS (27–29), there was only one report of T4SS effectors (i.e., from the plant pathogen *Xanthomonas citri*) killing or impeding other bacteria (i.e., *Chromobacterium violaceum* and *E. coli*) (55–57). Subsequently, the soil bacteria *Lysobacter enzymogenes* and *Pseudomonas putida* were found to also use their T4SSs to kill or otherwise impact environmental bacteria (58–61). Thus, the concept of T4SS as an agent of antibacterial activity, although new, is likely to grow in significance, given the presence of T4SSs in many other bacteria (62, 63). With this context, we now report the crystal structure of the previously defined *S. maltophilia* T4SS effector TfcB and the identification of an additional bactericidal effector (to be designated as TfdA), which is linked to nuclease activity.

## RESULTS

### Two-hybrid analysis identifies five more substrates of the *S. maltophilia* T4SS

**Fig 1:**
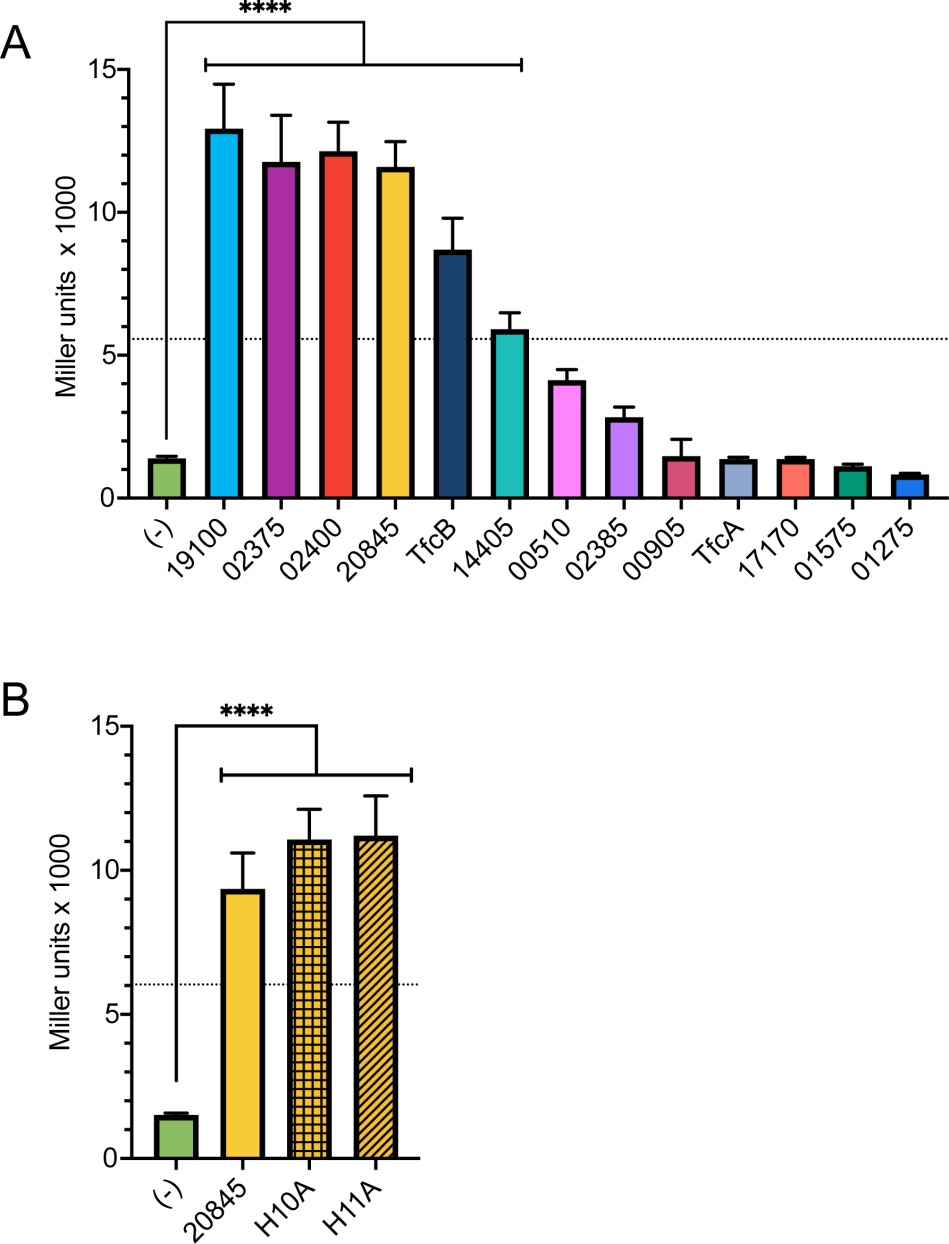
BACTH identification of *S. maltophilia* T4SS substrate interactions. (A) *E. coli* BTH101 containing both a “bait” plasmid expressing the *S. maltophilia* VirD4 protein fused to the T18 fragment of the adenylate cyclase of *Bordetella pertussis* (pUT18C-VirD4) and a “prey” plasmid derived from pKT25 that expresses one of the 11 putative T4SS effectors (denoted as 19100, 02375, 02400, 20845, 14405, 00510, 02385, 00905, 17170, 01575, or 01275), one of the known T4SS effectors (TfcB or TfcA), or no protein (−) fused to the T25 fragment of the *B. pertussis* adenylate cyclase were grown in selective media. Expression of an effector that directly binds the T4SS coupling protein results in the joining of the T18 and T25 fragments, reconstituting adenylate cyclase activity and increasing cAMP levels. Since BTH101 encodes a β-galactosidase gene that is under the control of a cAMP-dependent promoter, increased cAMP is detected in the presence of a β-galactosidase substrate o-nitrophenyl-beta-D-galactopyranoside (ONPG). Cleavage of ONPG was measured spectrophotometrically, and the levels of β-galactosidase activity were presented in Miller units. (B) *E. coli* BTH101 containing both a “bait” plasmid expressing the *S. maltophilia* immunity protein 20840 fused to the T18 fragment and a “prey” plasmid that expresses either the wild-type form of effector 20845 (20845), a mutant form of 20845 that has its histidine at residue-10 or residue-11 changed to an alanine (H10A, H11A), or no protein (−) fused to the T25 fragment was assessed for β-galactosidase activity as indicated in panel A. In panels A and B, data are presented as means and standard deviations, and the results are representative of at least three independent experiments. Asterisks indicate clones that had a level of β-galactosidase activity that was significantly more and at least four times greater (as denoted by the horizontal dotted lines) than that of the (−) control. Statistical analysis was performed using one-way analysis of variance (ANOVA) with Dunnett’s multiple comparison correction; ****, *P* < 0.0001.

Previously, we searched the *S. maltophilia* K279a genome for proteins that (i) had C-terminal features that are similar to those of known T4SS substrates, especially those from the closely related *Xanthomonas* genus, and (ii) were encoded next to an open reading frame (ORF) for a putative immunity protein (28). This analysis yielded 11 putative T4SS substrates, besides documented effectors TfcA and TfcB, and RT-PCR analysis indicated that all are expressed when K279a is grown alongside competing bacteria (28). As one approach to experimentally confirm which of these 11 are T4SS substrates, we sought to identify those that bind to *S. maltophilia* VirD4, the coupling protein that recognizes substrates in the cytoplasm and guides them to the inner membrane complex of the T4SS apparatus for eventual transit out of the bacterium (27, 63, 64). To that end, we utilized the bacterial adenylate cyclase-based two-hybrid (BACTH) assay (65, 66), as others had done to define effectors of T4SSs, T6SSs, and T7SSs (58, 67–74). Thus, in *E. coli*, we co-expressed a VirD4-encoding (high-copy-number) plasmid and a series of (low-copy-number) plasmids encoding the putative effectors and assayed for β-galactosidase activity from reconstituted adenylate cyclase. Similar to effector TfcB, five of the 11 possible effectors, encoded by ORFs RS19100 (smlt4012), RS02375 (smlt0500), RS02400 (smlt0505), RS20845 (smlt4383), and RS14405 (smlt3024), gave positive results in the BACTH assay, which meant a level of β-galactosidase that was at least four times greater than that of the negative control (Fig. 1A) (65). The remaining six encoded by ORFs RS00510 (smlt0113), RS02385 (smlt0502), RS00905 (smlt0193), RS17170 (smlt3607), RS01575 (smlt0332), and RS01275 (smlt0273) and effector TfcA did not result in significant β-galactosidase activity, suggesting weak or no binding of the protein products to VirD4 (Fig. 1A). However, it is possible for the BACTH assays to give false negatives (66, 75). Since one reason for false negatives is an improper ratio between the levels of bait and prey protein, we repeated the assay with VirD4 cloned into the low-copy vector and the putative effectors expressed from the high copy vector. However, those remaining proteins continued to show negative results (Fig. S1). Because the initial BACTH assays did reveal new T4SS substrates, we did not further interrogate the reasons for the lingering negative results. Among the confirmed VirD4 interaction partners, proteins “02375,” “02400,” and “14405” were annotated at National Center for Biotechnology Information (NCBI) as “hypothetical proteins,” whereas “19100” was annotated as a putative lipase and “20845” was annotated as a putative nuclease (28). We next focused on 20845 since its predicted nuclease activity was compatible with an antibacterial role, as exemplified by many T6SS effectors (76). In a variation of the BACTH assay, we documented that the wild-type (WT) form of 20845 and catalytic mutant forms of 20845, which will be more fully discussed in a later section, bind to the cognate immunity protein, “20840,” as expected for antibacterial effectors (Fig. 1B).

### Mutant lacking the T4SS substrate 20845 is impaired in killing bacterial competitors

**Fig 2:**
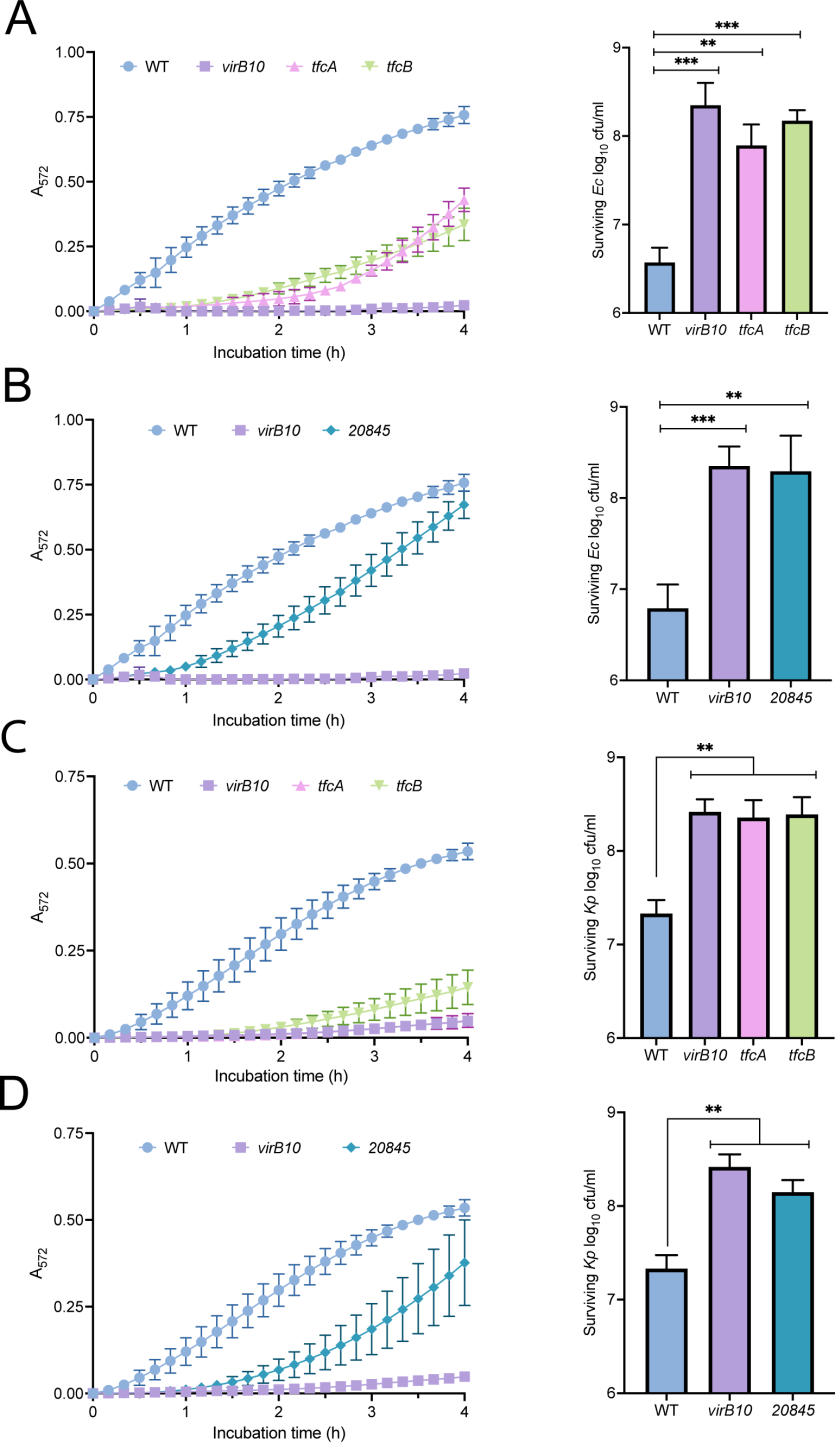
Bactericidal effect of *S. maltophilia* wild-type and T4SS mutants on *E. coli* and *K. pneumoniae*. (A–D) *S. maltophilia* K279a (WT), *virB10* mutant NUS15 (*virB10*), *tfcA* mutant NUS17 (*tfcA*), *tfcB* mutant NUS19 (*tfcB*), and 20845 mutant NUS24 (*20845*) were mixed (*n* = 6) at a 1:1 ratio (1–2 × 10^8^ CFU/mL each) with *E. coli* MG1655 (A, B) or *K. pneumoniae* DMDS (C, D), two strains that encode a β-galactosidase gene. The bacterial mixtures were incubated at 30°C in the presence of CPRG, a cell-impermeable substrate of β-galactosidase that changes color (yellow to red) when cleaved. The lysis of the target bacteria was detected by monitoring either increases in the absorbance of the cultures (A_572_) every 10 min over a 4-h period (A–D, left panels) or decreases in the numbers of surviving CFU of *E. coli* and *K. pneumoniae* as determined at *t* = 4 h (A–D, right panels). Data are presented as the means and standard deviations of results pooled from at least three independent experiments. In the left panels of A–D, differences between WT and the *virB10* mutant were evident from *t* = 50 min onward, with *P* < 0.05. In the left panels of A and C, differences between WT and the *tfcA* mutant and *tfcB* mutant emerged after 50 min, with *P* < 0.01. In the left panels of B and D, differences between WT and the *20845* mutants were observed between *t* = 50 min and *t* = 3 h, with *P* < 0.05. Statistical analysis was performed using two-way ANOVA with Dunnett’s multiple comparison correction. In the right panels of A –D, asterisks indicate when a mutant was different from WT, with **, *P* < 0.01; ***, *P* < 0.001.

**Fig 3:**
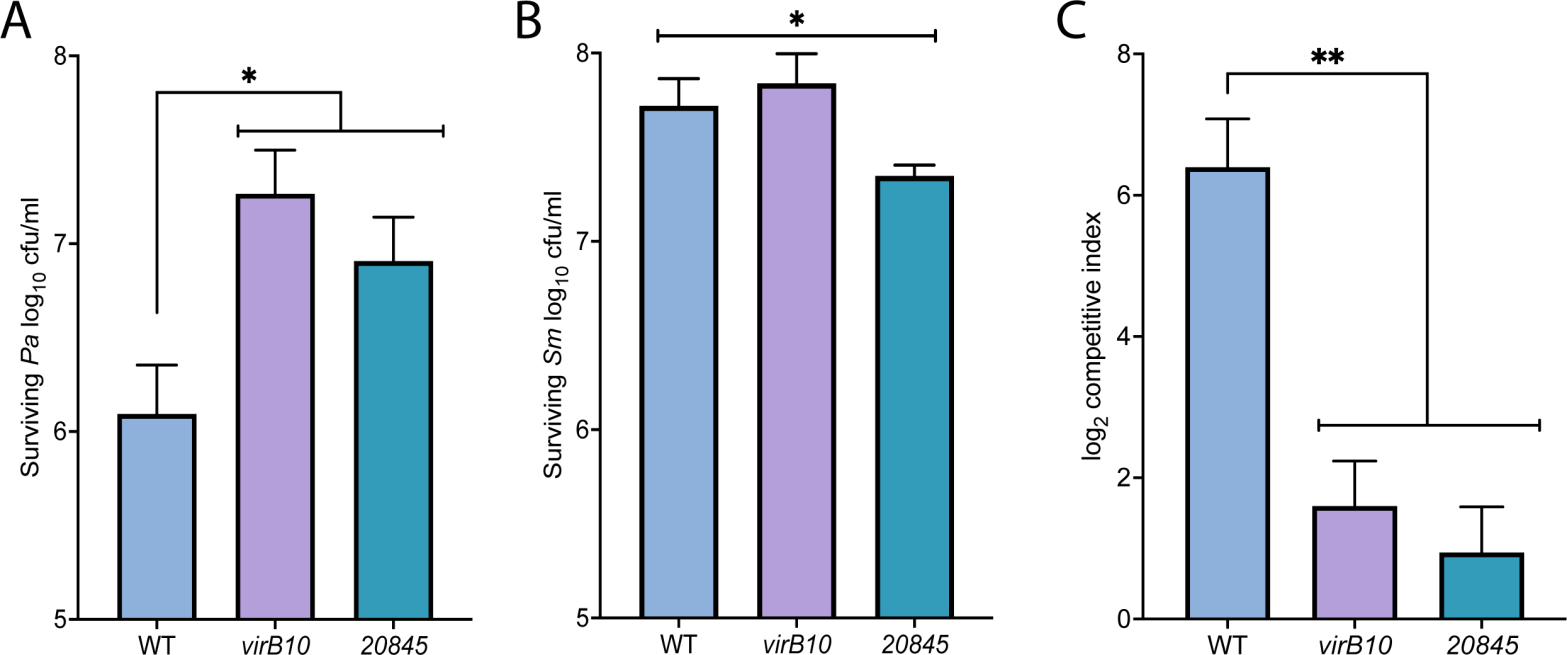
Bactericidal effect of *S. maltophilia* wild-type and 20845 mutants on *P. aeruginosa. S. maltophilia* (*Sm*) K279a (WT), *virB10* mutant NUS15 (*virB10*), and 20845 mutant NUS24 (*20845*) were mixed with *P. aeruginosa* (*Pa*) strain 7700 giving a (*Sm*/*Pa*) ratio of 1:1 (1–2 × 10^8^ CFU/mL each) and spotted onto a 0.2 µM nitrocellulose disk on the surface of an LB agar plate. After 4 h of incubation at 30°C, the numbers of *P. aeruginosa* (A) and *S. maltophilia* (B) bacteria remaining were determined by plating dilutions of the entire bacterial growth area on a selective medium. Based on the change in the ratio of *Sm*/*Pa* at *t* = 0 h vs. *t* = 4 h, the competitive index (CI) was calculated, where a positive log_2_ CI value indicates the *S. maltophilia* “attacker” outcompeting the target. For panels A–C, the data are presented as the means and standard deviations of results pooled from three independent experiments, with each experiment having three technical replicates. Asterisks indicate when incubations with the *virB10* and/or *20845* mutants resulted in bacterial recoveries (A–B) or CI (C) that were different from incubations WT *Sm*, with *, *P* < 0.05; **, *P* < 0.01.

To identify additional antibacterial factors associated with the *S. maltophilia* T4SS, we tested a mutant of *S. maltophilia* K279a that lacked 20845 for its ability to both lyse and outcompete heterologous bacteria. We mixed WT and mutant *S. maltophilia* with *E. coli* in a 1:1 ratio and monitored the release of cytoplasmic β-galactosidase from the target bacteria, analogous to past studies on effectors of T6SSs and T4SS effectors of *X. citri* (56, 57, 77–81). As expected (27–29), WT triggered a great deal of *E. coli* lysis during a 4 h co-incubation, whereas a *virB10* mutant of K279a lacking a functional T4SS showed no evidence of induced lysis (Fig. 2A, left). Providing another check on the assay, mutants missing the previously defined bactericidal effectors TfcA and TfcB (28) were also highly impaired for lysing competing bacteria (Fig. 2A, left). The mutants’ impaired bactericidal effects were confirmed when we assayed for surviving CFUs at the end of the 4-h time course (Fig. 2A, right). In the more sensitive chlorophenol red-β-d-galactopyranoside (CPRG)-based assay, the *tfcA* and *tfcB* mutants appeared to be less impaired than the *virB10* mutant (Fig. 2A, left), suggesting that there are additional bactericidal factors yet to be identified. Indeed, the mutant lacking the newly confirmed T4SS substrate 20845 was also impaired, showing a delay in the onset of target lysis (Fig. 2B, left). This result was affirmed when we assayed for surviving *E. coli* CFU (Fig. 2B, right). Similar to results obtained when examining T6SSs (78, 80, 82), the loss of TfcA, TfcB, or 20845 phenocopied the *virB10* mutant (i.e., the complete loss of the T4SS), when we measured CFU recovery after 4 h of co-incubation. This suggests that the three effectors act in a concerted manner and that each of them is needed for maximal killing to occur, at least in this assay condition. Next, when *Klebsiella pneumoniae* (*Kp*) strain DMDS was used as the competitor, we saw for the first time that the *S. maltophilia* T4SS and effectors TfcA and TfcB can kill a *K. pneumoniae* strain (Fig. 2C). Subsequent trials indicated that T4SS substrate 20845 is also toxic toward *K. pneumoniae* (Fig. 2D). Supporting the conclusions regarding the effects of 20845 on *E. coli and K. pneumoniae*, the 20845 mutant, like the *virB10* mutant, *tfcA* mutant, and *tfcB* mutant, survived similarly to WT over the 4-h co-incubation (Fig. S2). Finally, we examined *S. maltophilia* competition against *P. aeruginosa* strain 7700 by co-incubating the bacteria on Luria Bertani (LB) agar and monitoring changes in their CFU ratio over time (27, 28). The 20845 mutant was once again impaired for killing, exhibiting a loss in competitive index that mirrored that of the *virB10* mutant (Fig. 3). Together, these data indicated that 20845 likely contributes to the bactericidal activity of the *S. maltophilia* T4SS against a range of Gram-negative targets.

### Effector 20845 is sufficient to kill competing bacteria

**Fig 4:**
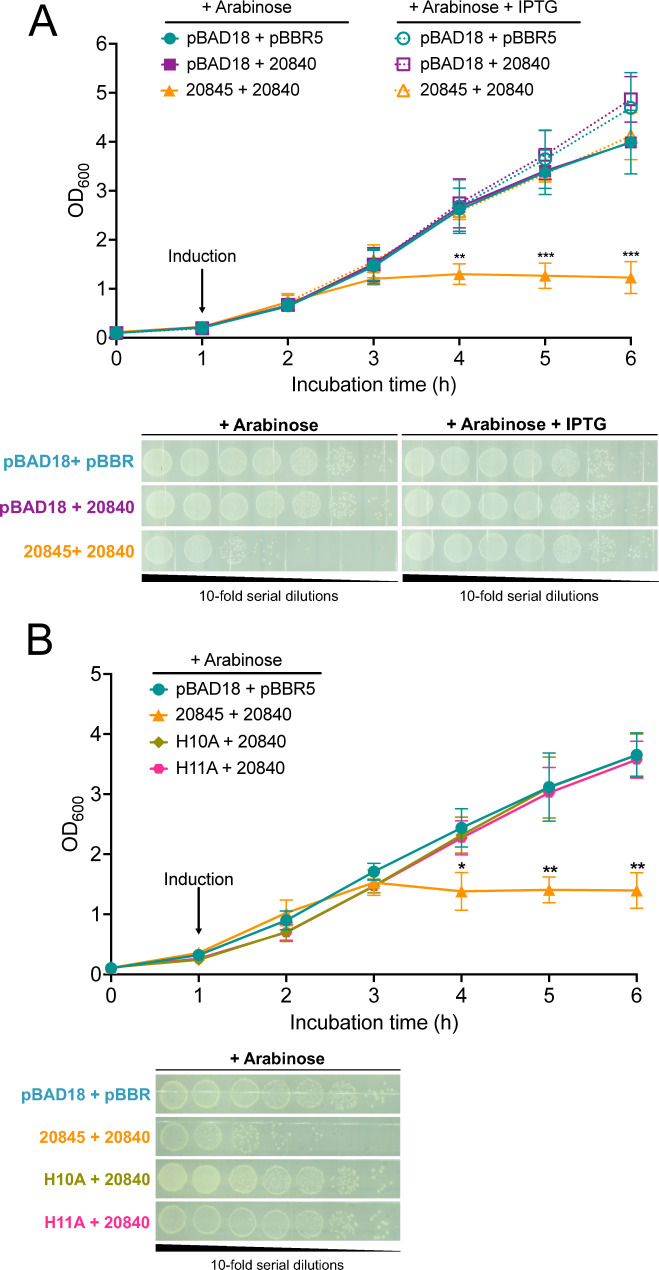
Effect of the cloned T4SS effector 20845 on the viability of heterologous bacteria. (A) *E. coli* DH5α containing either (i) vectors pBAD18 and pBBR1MCS-5 (pBAD18 + pBBR5), (ii) pBAD18 and the 20840 immunity protein gene cloned into pBBR1MCS-5 (pBAD18 + 20840), or (iii) the wild-type 20845 gene cloned into pBAD18 and the 20840 immunity protein gene in pBBR1MCS-5 (20845 + 20840) were inoculated into LB broth that contained added glucose, which promoted repression of the 20845 gene. After 1 h of incubation (arrow), the medium was switched to LB broth that contained either added arabinose and IPTG to induce expression of both 20840 and 20845 or added arabinose to induce expression of only 20845. Subsequent bacterial survival at 37°C was assessed by measuring the cultures’ OD_600_ hourly (upper panel) and plating 10-fold serial dilutions of the cultures onto LB agar at 6 h (lower panel). (B) *E. coli* DH5α containing either (i) pBAD18 + pBBR5, (ii) 20845 + 20840, (iii) the H10A mutant form of the 20845 gene in pBAD18 and the 20840 gene in pBBR1MCS-5 (H10A + 20840), or (iv) the H11A mutant form of the 20845 gene in pBAD18 and the 20840 gene in pBBR1MCS-5 (H11A + 20840) were inoculated into LB broth that contained added glucose. At the 1-h time point (arrow), arabinose was added to induce the expression of 20845. Subsequent bacterial growth was assessed by measuring the OD_600_ of the cultures hourly (upper panel) and CFU (lower panel). For panels A and B, the data are presented as the means and standard deviations of results pooled from three independent experiments. Asterisks indicate points at which a given clone’s growth was significantly reduced compared with all the other clones tested, with *, *P* < 0.05; **, *P* < 0.01; ***, *P* < 0.001.

Pursuing further evidence for the bactericidal effect of 20845, we tested if expression of this effector alone was sufficient to alter the viability of a competitor. It was immediately evident that *E. coli* containing the 20845 ORF cloned into vector pBAD18 was severely impaired for growth whether or not arabinose-mediated induction of the ORF was applied. To document the impact of 20845 more clearly, we generated a new *E. coli* strain that expressed its cognate immunity protein 20840 under the control of an isopropyl-β-D-thiogalactopyranoside (IPTG)-inducible promoter in vector pBBR1MCS-5 and introduced into that strain pBAD18 carrying an arabinose-inducible 20845 gene. *E. coli* induced to simultaneously express effector 20845 and immunity protein 20840 grew similarly to *E. coli* expressing just the immunity protein or the empty vectors (Fig. 4A). Most importantly, when *E. coli* was induced to express 20845 in the absence of co-induction of 20840, growth was severely impaired as evidenced by large reductions in the OD of and bacterial recovery from the cultures (Fig. 4A). These results, combined with the mutant data, documented that effector 20845 promotes the bactericidal activity of the *S. maltophilia* T4SS.

### Effector 20845 confers a bactericidal DNase activity

**Fig 5:**
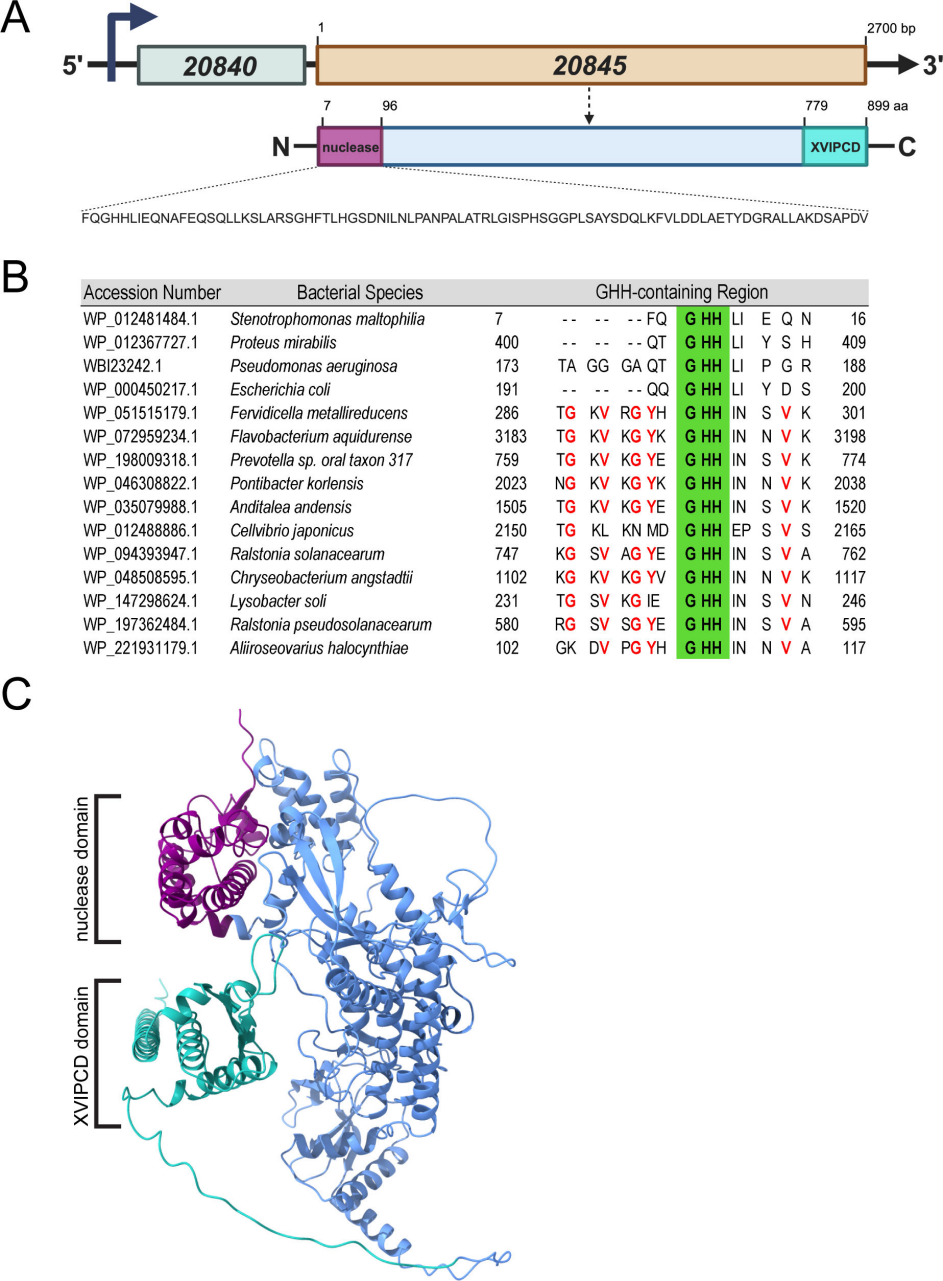
Genetic locus, predicted structure, and GHH-containing region of the 20845 effector. (A) Depicted at the top of this figure is the chromosomal locus of strain K279a that encodes the 20845 effector and its cognate immunity protein 20840. The gene map given below is a representation of the 20845 protein, showing its N-terminal nuclease domain, large central region, and C-terminal XVIPCD. The amino acid residue numbers that mark the beginnings and ends of these parts of 20845 are also provided. Finally, under the protein depiction is the sequence of the predicted nuclease domain. (B) The section of 20845’s N-terminus that contains the GHH-containing catalytic site is presented in alignment with similar regions from a subset of the previously identified GHH-containing nucleases from bacteria. For each bacterial entry, the numbers to the left and right of the amino acid sequences correspond to the position of the GHH-containing region within the overall protein. The defining GHH residues are in green highlight, and other residues that are conserved in a subset of the proteins are in red font. (C) Predicted 3D structure of 20845 as determined by AlphaFold-2. The protein’s N-terminal nuclease domain, central region, and XVIPCD are denoted with different colors.

Having confirmed an antibacterial role for 20845, we began to investigate the protein’s structure function and the existence of homologs or related proteins among microbial species. In addition to having a C-terminal T4SS secretion signal known as the *Xanthomonas* VirD4-interacting protein conserved domain (XVIPCD), 20845 was notable for its large size, that is, 899 amino acids (aa) and predicted molecular weight of 96.1 kDa (Fig. 5A). Compatible with its prior annotation at NCBI (28), comparisons to the protein database indicated that the N-terminus of 20845 has a motif corresponding to the catalytic site present in bacterial GHH nucleases, a minimally characterized family within the HNH/Endo VII superfamily of nucleases (Fig. 5A and B (83–87). Between this putative enzymatic domain and the secretory XVIPCD, there was a large region that did not exhibit similarity to any (portion of a) characterized protein (Fig. 5A). When we used Alpha-Fold software (88) to predict the 3D structure of the 20845, the N-terminal nuclease domain and C-terminal XVIPCD were well separated from each other and distinctly positioned from the large central region of unknown function (Fig. 5C). BLASTP searches indicated that close homologs of 20845 (i.e., hypothetical proteins with >90% aa identity across the entire length of the protein) or related proteins (i.e., 28% to 41% aa identity across 51% to 85% of the protein) are encoded by 46/60 of the other *S. maltophilia* strains examined (Table S1). This analysis also revealed 20845-related proteins in 10/24 other sequenced species of *Stenotrophomonas* (Table S2). Outside of *Stenotrophomonas*, 20845 had its most striking homologs (i.e., hypothetical proteins with 50% to 99% identity across the entire length of the protein) in strains of the unrelated bacteria *Pseudomonas cichorii, Acinetobacter baumannii, K. pneumoniae,* and *Raoultella ornithinolytica*, the fungus *Knufia peltigerae*, and several species of *Xanthomonas*, a *Stenotrophomonas*-related genus in the order *Xanthomonadales* (Table S3). Protein 20845 also had strong similarity (i.e., 30% to 65% aa-identity across its central region and XVIPCD) to other hypothetical proteins mainly from other species of *Xanthomonas* (Table S3). The 20845 protein displayed similarities in either its N-terminus alone or across its N-terminus and central region to putative GHH nucleases of *Xanthomonas* spp., *K. pneumoniae*, and *Neisseria gonorrhoeae* that, to our knowledge, had not been previously reported (Table S3). Other lower-level matches for 20845 resulted from sharing similarity within (only) the XVIPCD to hypothetical proteins from *Xanthomonas* and other members of the *Xanthomonadales* (e.g., *Lysobacter, Thermomonas*, *Vulcaniibacterium*) (Table S3). The remaining matches resulted from 20845’s central region showing similarity to hypothetical proteins that were mainly in *Xanthomonadales* and included zeta toxin family members (Table S3). Given these BLASTP results, 20845 appeared representative of a novel form of effector/nuclease.

**Fig 6:**
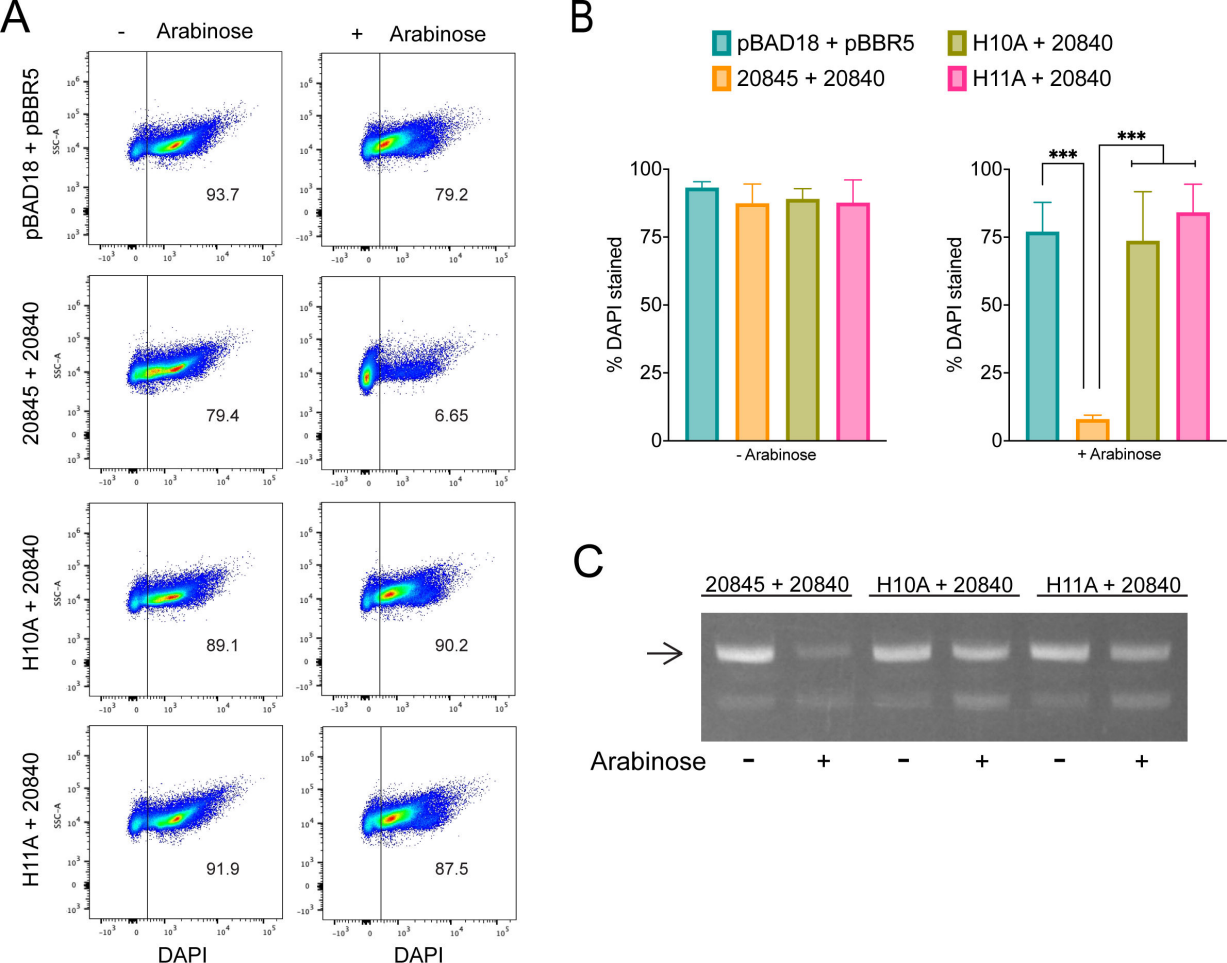
DNase activity associated with wild-type and mutant forms of the T4SS effector 20845. *E. coli* DH5α containing either (i) pBAD18 and pBBR1MCS-5 (pBAD18 + pBBR5), (ii) the wild-type 20845 gene in pBAD18 and the 20840 gene in pBBR1MCS-5 (20845 + 20840), (iii) the H10A mutant form of the 20845 gene in pBAD18 and the 20840 gene in pBBR1MCS-5 (H10A + 20840), or (iv) the H11A mutant form of the 20845 gene in pBAD18 and the 20840 gene in pBBR1MCS-5 (H11A + 20840) were grown for 3 h at 37°C in LB broth that either lacked added arabinose to limit the expression of the 20845 gene (− arabinose) or contained added arabinose to induce expression of the 20845 gene (+ arabinose). Aliquots taken from each sample were stained with DAPI and analyzed by flow cytometry, in which 10^5^ events were counted, and the percentage of DAPI-stained cells was calculated. (A) The presented dot plots are representative of the flow cytometry output from three independent trials. (B) The presented bar graphs report the means and standard deviations of %DAPI-stained cells for each strain grown without (left panel) or with arabinose (right panel) pooled from the three experiments. Asterisks indicate differences between the samples, with ***, *P* < 0.001. (C) Aliquots from the above-described bacterial suspensions were lysed, and equal amounts of recovered DNA were electrophoresed through an agarose gel and stained with ethidium bromide. The presented image highlights the varying levels of the main form of the pBAD18-based plasmids (arrow) and is representative of the results from at least two independent trials. The smaller pBBR1MCS-5-based plasmids were less apparent likely due to their lower copy number.

To determine if the bactericidal activity of 20845 is associated with its N-terminal nuclease motif, we modified the genetic codons for histidine-10 and -11 (Fig. 5B) to alanine in the cloned 20845 gene (Fig. 5B) and then tested the ability of mutant variants of the expressed protein (“H10A” and “H11A”) to confer toxicity in *E. coli*. BACTH analysis confirmed that the two amino acid-substituted forms of 20845 bind to the immunity protein 20840 similar to wild-type 20845 (Fig. 1B), indicating that they are properly folded and expressed to similar levels in *E. coli*. Importantly, neither mutant form of 20845 triggered the growth arrest and reduction in CFU that were associated with the wild-type form of 20845 (Fig. 4B), confirming that the site containing H10 and H11 is required for toxicity and suggesting that 20845 acts as a nuclease. Utilizing an approach that has been applied to the study of T6SS DNases (67, 89), we used flow cytometry to monitor DNA integrity in *E. coli* clones expressing 20845. When *E. coli* was induced to express 20845 in the absence of co-induction of the immunity protein 20840, there was a marked decrease in DNA content (i.e., loss of 4’,6-diamidino-2-phenylindole [DAPI])-staining), compared with when the clone was repressed for 20845 expression (Fig. 6A and B). This detrimental effect was not seen for clones expressing the H10A and H11A mutant forms of 20845 (Fig. 6A and B). The wild-type 20845, but not its H10A and H11A variants, had DNase activity that was further indicated when the *E. coli* clones were examined for degradation of endogenous plasmid by ethidium bromide staining (Fig. 6C), akin to past studies of T6SS nucleases (82, 89–92). Together, these data indicated that 20845 is a DNase and that its nuclease activity promotes the bactericidal effect of the protein. Thus, we named 20845 and its gene as TfdA and *tfdA*, for type four secretion system DNase effector A.

### Structural determination of TfcB

**Fig 7:**
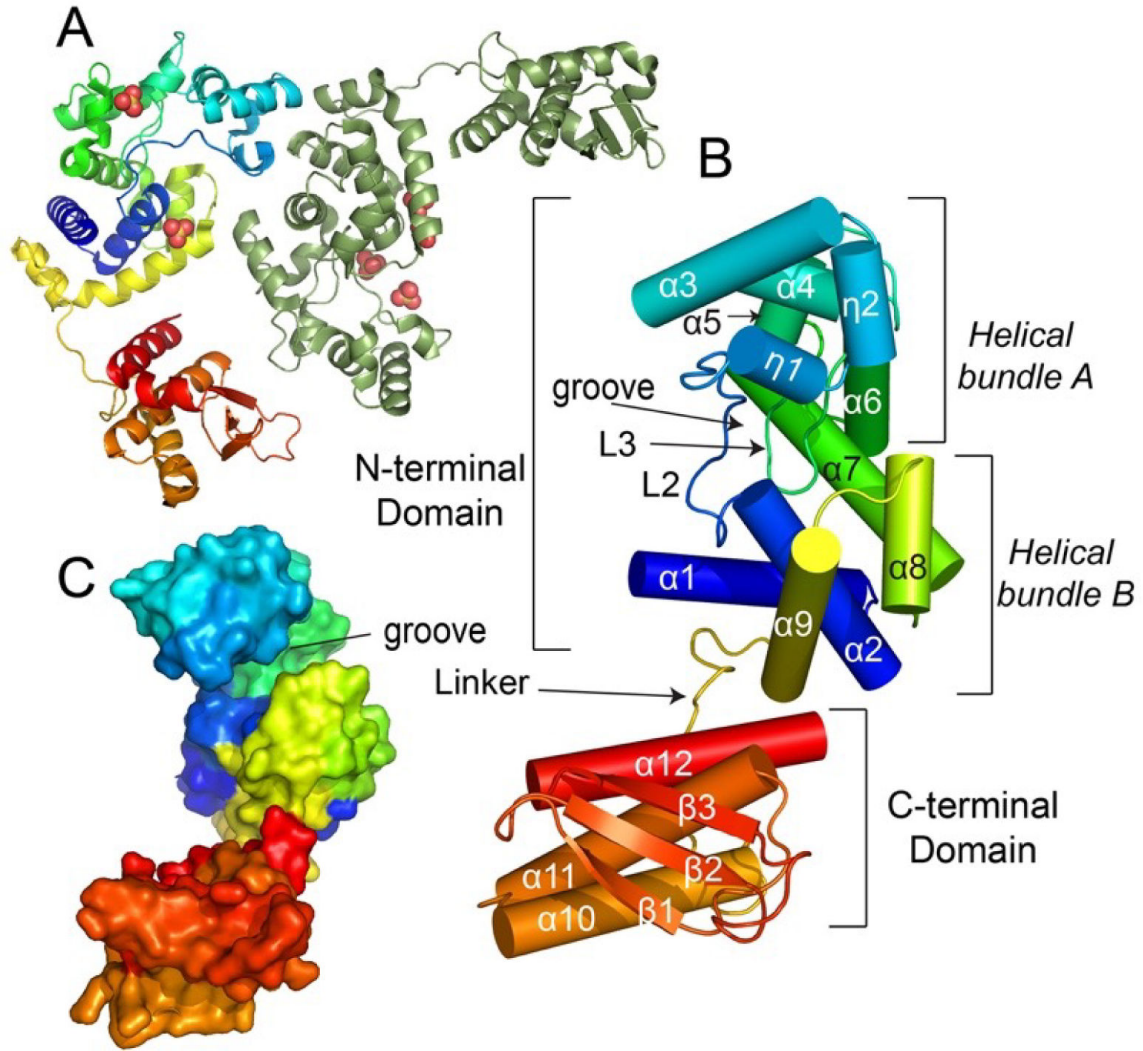
Overall structure of the *S. maltophilia* T4SS effector TfcB (PDB 7r6s). (A) Ribbon diagram of two molecules of TfcB in the asymmetric unit. One of the molecules is colored as a rainbow. The structure also shows the presence of sulfate ions (red trimeric balls). (B) The structure is comprised of two domains connected by a linker. The N-terminal domain (residues 1–200) consists of α helices 1 to 9, whereas the C-terminal domain (residues 219–316) consists of α helices 10 to 12 and β strands 1 to 3. A groove is formed between two helical bundle A (η1–η2, α3–α6) and helical bundle B (α1, α2, α8, and α9). Both the bundles are connected by α7 and L2. (C) Surface representation of TfcB depicting the groove.

To further characterize the defined effectors of the *S. maltophilia* T4SS, we submitted them for structure determination to the Center for Structural Biology of Infectious Diseases (CSBID). As a result, we determined the structure of TfcB to 1.9 Å. The structure, deposited in the Research Collaboratory for Structural Bioinformatics (RCSB) Protein Data Bank (PDB) under the PDB code 7r6s, includes residues 1–316 with the last 18 aa disordered and thus missing from the structure. The data and refinement statistics are listed in Table S4. There are two molecules of TfcB in the asymmetric unit (Fig. 7A). Each protomer consists of 12 α helices, 2–3_10_ helices, and 3 β sheets and forms two domains. The N-terminal domain is composed of two helical bundles. Helical bundle A (η1–η2, α3–α6) forms an apical head-like region that is linked to bundle B (α1, α2, α8, and α9) by long helix α7 and loop 2 (L2 connects α2 and η1). These helical bundles connect to create a cleft or concave groove that consists primarily of residues from L2 and loop 3 (L3) that connects α5 and α6 and protrudes down into this groove. (Fig. 7B and C). The C-terminal domain is composed of a single helical bundle (α10, α11, and α12) adjacent to a three-stranded, anti-parallel β-sheet (Fig. 7B). The N- and C-terminal domains are connected by a long loop/linker. These results represent the first structural determination for an antibacterial effector from a T4SS.

### Structural homology of TfcB to proteins of known function

**Fig 8:**
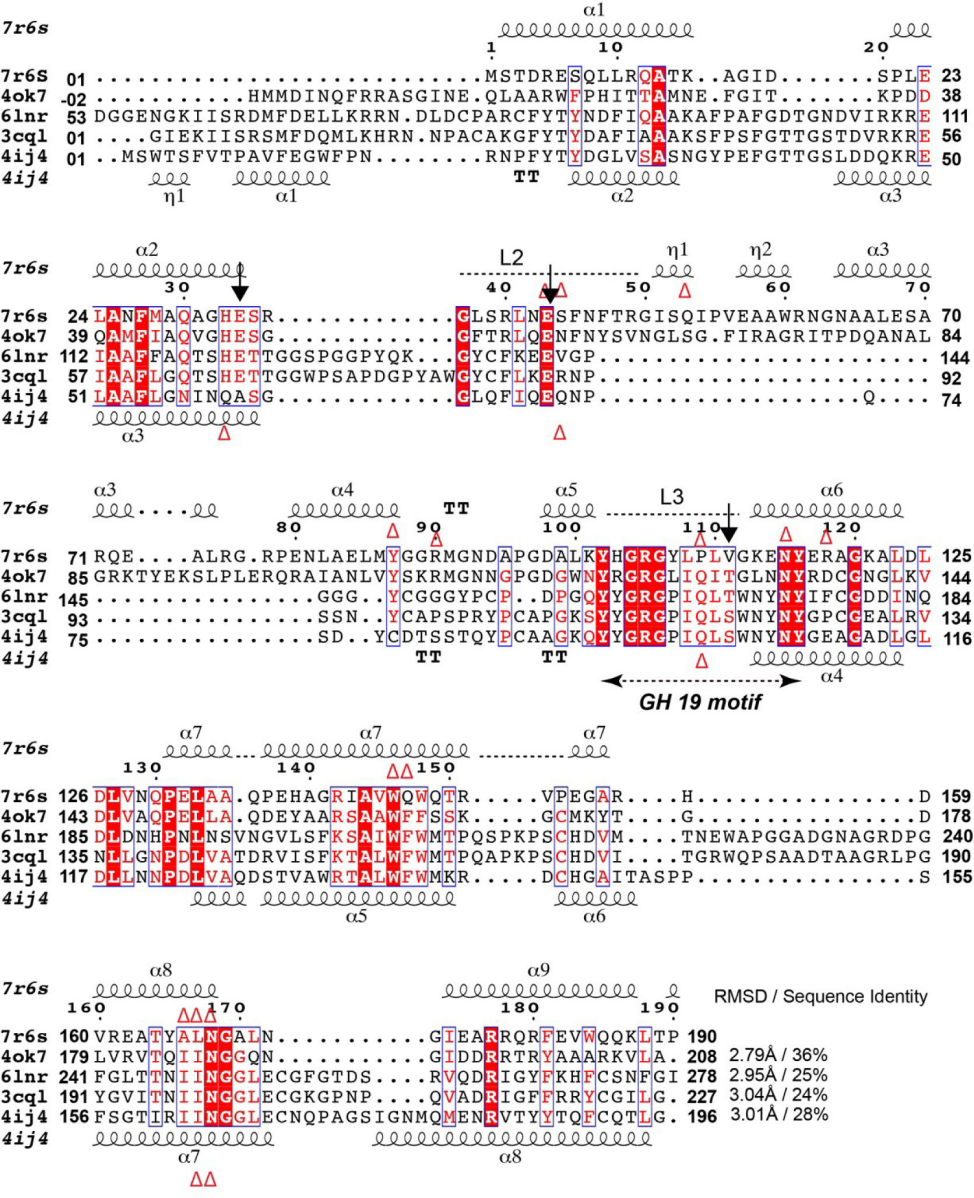
Alignment of the N-terminus of TfcB with the N-termini of GH family 19 chitinases. Structure-based sequence alignment of TfcB (7r6s) with enzymes from bacteriophage SPN1S (4ok7), the plants *S. glauca* (6lnr) and *C. papaya* (3cql), and the moss *B. coronatum* (4ij4). The numbering and secondary structure at the top correspond to TfcB. The bottom secondary structure corresponds to 4ij4. Conserved GH-19 catalytic residues are labeled by black arrows. Structure 4ij4 is a catalytically inactive variant E61A, and hence, glutamic acid residue is not present. Red triangles represent residues involved in interaction with the substrate (GlcNAc)_4_.

We used the Distance matrix Alignment (DALI) server (93) to probe the PDB for structural homologs of TfcB and predict the function of this protein. When the full-length structure was used, we identified several enzymes of the glycosyl hydrolase (GH) Family 19 chitinases (GH-19) (94) as being similar. This group included the endolysin of the *Salmonella typhimurium* bacteriophage SPN1S (PDB 4ok7, *Z*-score of 16.8, RMSD of 2.7) (95), a chitinase from the moss *Bryum coronatum* (PDB 4ij4, *Z*-score of 13.7, RMSD of 2.7) (96), and chitinase from the papaya plant *Carica papaya* (PDB 3cql, Z-score 11.9, RMSD 2.9) (94). To better identify which TfcB domain is more closely related to these enzymes, we submitted the N- and C-terminal domains separately. A search using only the N-terminal domain returned several GH-19 chitinases, with the most similar structures being the phage SPN1S endolysin (PDB 4ok7, *Z*-score 20.0, root mean square deviation [RMSD] of 2.7) (95) and the chitinase from plant *Simarouba glauca* (PDB 6lnr, *Z*-score 12.0, RMSD of 3.1) (97). At the primary sequence level, our protein shares 36%, 25%, 28%, and 24% identity with the SPN1S endolysin and the chitinases from *S. glauca*, *B. coronatum*, and *C. papaya*, respectively (Fig. 8). Thus, TfcB is comprised of a fold similar to that of characterized GH-19 chitinases/endolysin.

Structural comparisons of TfcB to GH-19 enzymes revealed that both the N-terminal domain of TfcB and the phage endolysin harbor an apical head-like structure (η1–η2, α3–α6), whereas the plant chitinases from *C. papaya*, *B. coronatum*, and *S. glauca* do not (Fig. S3A through D). In previously characterized GH-19 enzymes, the loops (L2 and L3) contribute to the formation of a groove. In plant chitinases, these grooves are bound by homopolymers of N-acetyl-d-glucosamine (GlcNAc), e.g., PDB 4ij4 and 3cql. The presence of the apical head-like bundle and the orientations of L2 and L3 observed in TfcB and SPN1S result in a deeper putative substrate binding groove compared with the plant chitinases. The volume of the groove region estimated by CASTp server is higher for TfcB (700.2 Å^3^) and the phage protein (953.8 Å^3^) compared with the plant chitinases of *S. glauca* (83.76 Å^3^), *B. coronatum* (227.1 Å^3^), and *C. papaya* (403.9 Å^3^). The suspected substrate for the bacteriophage SPN1S endolysin is peptidoglycan, and it does not act on homopolymers of GlcNAc (95). Furthermore, the SPN1S protein’s apical head-like domain is important for interactions with peptidoglycan (95). These analyses demonstrate that TfcB shares significant structural homology with GH-19 enzymes and may target a component of bacterial cell walls.

**Fig 9:**
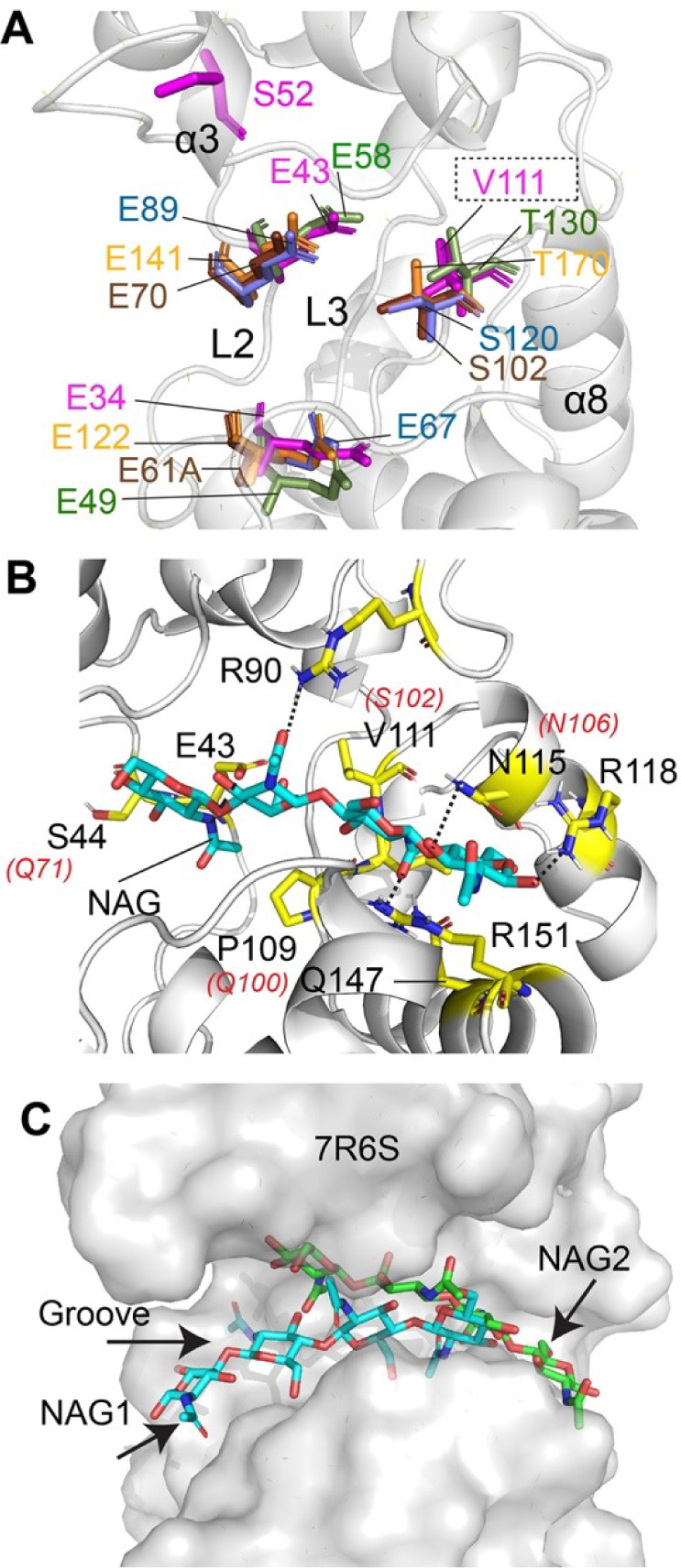
Catalytic site of TfcB and other GH family 19 chitinase. (A) Overlay of TfcB with GH family 19 chitinase from bacteriophage SPN1S (4ok7, green), the plants *S. glauca* (6lnr, yellow) and *C. papaya* (3cql, blue), and the moss *B. coronatum* (4ij4, brown) shows conservation of two catalytic Glu residues. For 4ij4, E61 has been substituted by mutagenesis with alanine in this structure. Note the third catalytic Ser/Thr residue is replaced by Val111 in TfcB. The only Ser residue close to the two putative catalytic Glu residues is from α3 Ser52. (B) Structure of TfcB (7r6s) with residues involved in interaction with the substrate (GlcNAc)_4_ shown in yellow based on docking using a haddock server. Similar residues that bind (GlcNAc)_4_ from *B. coronatum* (4ij4) are italicized and highlighted in red. (C) Orientation of (GlcNAc)_4_ (NAG2, green) inside the groove of TfcB based on docking using the Haddock server. The structure is superimposed with the position of (GlcNAc)_4_ (NAG1, blue) from PDB 4ij4.

Previous work on GH-19 enzymes identified a conserved catalytic triad (Glu-Glu-Ser/Thr) for which the critical role of each residue in substrate hydrolysis has been explained in the *C. papaya* and *B. coronatum* chitinases (94, 96). In addition, a 15-residue conserved motif (Y-[FHY]-G-R-G-[AP]-x-Q-[IL]-[ST]-[FHYW]-[HN]-[FY]-N-Y) that contributes to substrate binding was also characterized in the *C. papaya* chitinase and can be found in closely related enzymes (94). A structural sequence alignment of TfcB with these GH-19 enzymes revealed that residues Glu34 (α2) and Glu43 (L2) are conserved in TfcB (Fig. 8). These putative catalytic residues are positioned in the groove formed by L2 and L3 in our structure, and this groove is similarly positioned in other GH-19 chitinases (Fig. 9; Fig. S3). Furthermore, the 15-residue substrate binding conserved motif spans residues Tyr102 to Tyr116 in the L3 region of our structure (Fig. 8). However, several residues in this motif are distinct from other GH-19 chitinases, including Tyr107, Pro109, Val111, Gly112, Lys113, and Glu114. Structural overlay of TfcB to that of these GH-19 enzymes showed that residues Glu34 and Glu43 of TfcB align with the catalytic Glu residues of the well-characterized chitinases (Fig. 9A). However, Val111 aligns with the Ser/Thr residue of the conserved GH-19 catalytic triad (Fig. 8 and 9A). In the TfcB structure, the closest Ser52 (α3) residue to the catalytic Glu34 and Glu43 resides 16–19 Å from the putative active site. The Ser-to-Val substitution in the catalytic triad of TfcB and numerous non-conserved amino-acid substitutions in the substrate binding motif suggested that this effector possibly targets a substrate other than GlcNAc or chitin.

To predict the binding site for chitin on TfcB, we performed unbiased docking of four molecules of GlcNAc [(GlcNAc)_4_] to the TfcB structure using the independent docking servers Haddock (98), H-dock (99), and E-dock (100). Each of these servers uses different algorithms, and docking was done without any structural constraints. All three servers docked (GlcNAc)_4_ at a similar region of TfcB with subtle differences in the orientation (Fig. 9B; Fig. S4). Residues of TfcB involved in hydrogen bonding and hydrophobic interactions with (GlcNAc)_4_ are mostly from the L2 region (Arg40, Glu43, and Ser44), L3 region (Pro109, Leu110, and Val111), α8 (Glu114, Asn115, and Arg118), and aromatic residues including Phe47, Tyr87, Trp146, and Tyr165 (Fig. 9; Fig. S5). Binding residues from L3 span the conserved 15-residue GH-19 chitinases motif, whereas the other interacting residues also show conservation to the GH-19 chitinases (Fig. 8). Furthermore, an overlay of the model of TfcB in complex with (GlcNAc)_4_ to *B. coronatum* chitinase bound to GlcNAc (PDB 4ij4) (Fig. 9C) highlights the differences in binding of GlcNAc between these proteins inside the groove. As evident for the *B. coronatum* protein (Fig. 9C), the GlcNAc molecules primarily bind to the anterior part of the groove and extend all the way to the middle of the groove, whereas for TfcB GlcNAc binds from the middle of the groove and extends all the way backward toward the groove. To summarize, the variation in depth of the groove as well as modifications in amino acids within the conserved 15 -residue GH-19 chitinases motif for TfcB compared with other chitinases may result in differences in substrate/target preferences or substrate/target orientation for its activity.

**Fig 10:**
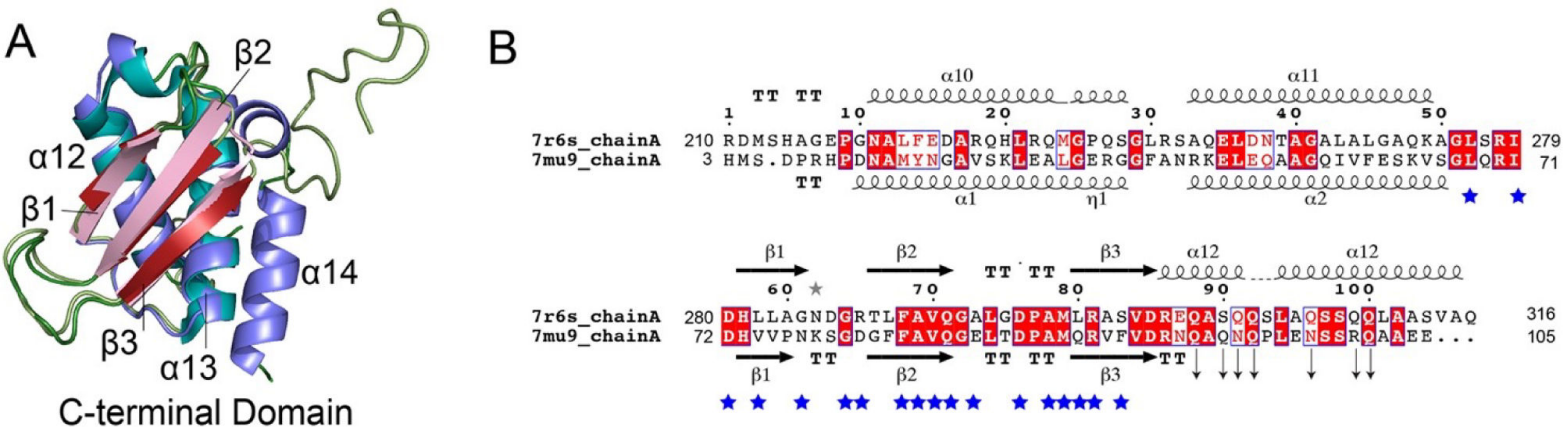
Structural similarity of the C-terminal domain of TfcB with the XVIPCD from *X. citri*. (A) Structural alignment of the C-terminal domain of TfcB with the C-terminus of X-Tfe (XAC2609) from *X. citri* (7mu9). TfcB is colored as α helices (blue) and β strands (pink) with the X-Tfe structure α helices (teal) and β strands (red). TfcB has a C-terminal α helix (α14), which remains unstructured in X-Tfe (XAC2609). (B) Structure-based sequence alignment of TfcB (7r6s, amino acids 210–316) with X-Tfe (XAC2609) (7mu9, amino acids 3–105). Blue asterisks denote the amino acids of X-Tfe (XAC2609) that are known to interact with VirD4, whereas the arrows mark charged residues required for secretion.

Comparison of the C-terminal domain of TfcB using the DALI server (93) revealed significant structural similarity to the XVIPCD found in the C-terminal region of the *X. citri* T4SS effector X-Tfe(XAC2609) (PDB 7mu9, Z-score 11.8, RMSD 2.1 Å) (81) (Fig. 10). The N-terminal region of the XVIPCD interacts with the alpha-helical domain of the T4SS ATPase VirD4, whereas the C-terminal domain is rich in charged glutamine residues required for secretion of the effector. Alpha helices α12 and α13 and the anti-parallel β-sheet of the TfcB C-terminal domain structurally overlap with the XVIPCD structure (Fig. 10A). However, in our structure, an extra α helix (α14) is fully resolved, whereas this region is unstructured in the previously defined XVIPCD (81). Several residues in the XVIPCD were identified as important for interactions with VirD4 and contribute to effector recognition and translocation through the T4SS apparatus (81). Structural sequence alignment of the *X. citri* XVIPCD with TfcB (Fig. 10B) highlights conserved residues, suggesting that this domain is also involved in translocation of TfcB.

## DISCUSSION

With the present characterization of TfdA, there are now three documented bactericidal effectors associated with the *S. maltophilia* VirB/VirD4 T4SS, i.e., TfdA, TfcA, and TfcB. Presumably, having multiple bactericidal effectors helps *S. maltophilia* to effectively kill a range of competitors. Based on *in silico* analysis and multiple forms of experimental evidence, the antibacterial effect of TfdA is due, at least partly, to DNase activity mapping to the protein’s N-terminal GHH-containing domain. Thus, we posit that, following its injection into the periplasm of target bacteria by the T4SS apparatus, TfdA crosses the competitor’s inner membrane and acts on nucleic acid in the cytoplasm. Another question for future inquiry is whether TfdA degrades DNA in a specific or non-specific manner. That a secreted nuclease promotes interbacterial killing/competition is not a new concept, e.g., there are examples of such effectors from T6SSs of species of *Acinetobacter, Aeromonas, Agrobacterium, Bacteroides, Burkholderia, Dickeya, Escherichia, Myxococcus, Neisseria, Pectobacterium, Phocaeicola, Proteus, Pseudomonas, Salmonella, Serratia, Vibrio*, and *Yersinia* and a T7SS of *Staphylococcus* (67, 68, 71, 73, 82, 85, 89–92, 101–113). However, based on BLASTP results, TfdA is overall unrelated to these previously characterized nucleases, including PefD of *Proteus mirabilis*, the only other bacterial GHH-nuclease that has been studied beyond *in silico* classification (Fig. 5B). Indeed, the only significant homologs to TfdA were hypothetical proteins from other species of *Stenotrophomonas* and *Xanthomonas* and uncharacterized proteins from *P. cichorii, R. ornithinolytica*, *A. baumannii, K. pneumoniae,* and the fungus *K. peltigerae* (Table S3). Thus, TfdA is representative of a new class of broadly distributed nucleases that may include more antibacterial effectors. TfdA also stands out for its large 899-aa size, which is mostly comprised of a ~ 683 aa central region (between the N-terminal nuclease domain and C-terminal secretion domain) that does not show similarity to any characterized domains. It is possible that this uncharacterized region may encode another, toxic activity. Along those lines, it is worth restating that the *S. maltophilia* T4SS also has effects on mammalian targets, for example, promoting apoptosis in macrophages (27). Thus, among other things, it will be interesting to ascertain the impact of TfdA (and other *S. maltophilia* T4SS substrates) on eukaryotic cells. Unlike most of the defined and presumptive substrates of the *S. maltophilia* T4SS (28), TfdA was present in only 77% of *S. maltophilia* strains and 42% of *Stenotrophomonas* species. This, coupled with the fact that nearly identical proteins were detected in many species of *Xanthomonas* and several other unrelated genera, suggests that TfdA might be acquired by horizontal gene transfer.

The present study has also advanced our understanding of T4SSs and the *S. maltophilia* T4SS, particularly by determining the structure of the previously identified bactericidal effector TfcB (28). From this structure, we can now unquestionably classify TfcB as a novel member of the GH-19 family of chitinase-like molecules. TfcB’s N-terminal domain consists of a motif with a fold similar to GH-19 chitinases with an architecture most similar to the endolysin from bacteriophage SPN1S, whereas the C-terminal domain of the protein is structurally similar to the T4SS effector recognition signal domain from *X. citri* (81). Our docking studies predict that TfcB can bind to (GlcNAc)_4_ and chitin but in a different orientation compared with known chitinases. However, in terms of explaining the bactericidal effect of TfcB, the structural data are most compatible with the protein recognizing different compositions of peptidoglycan. However, it is also possible that the bacterial target is another type of GlcNAc-containing molecule(s). These alternate reactions might potentially utilize serine Ser52 along with glutamates Glu34 and Glu43. That TfcB appears to also bind chitin raises the possibility that TfcB and the *S. maltophilia* T4SS might also target chitin-containing organisms in nature, such as fungi.

Although we mainly focused on the further characterization of TfdA and TfcB, the BACTH analysis that began the study suggested that proteins 14405, 19100, 02375, and 02400 are additional substrates of the *S. maltophilia* T4SS. Like TfcA before it (28), 19100 is annotated as a lipase, and therefore, we predict that this protein will prove to have bactericidal activity, especially when examined using the CPRG-based killing assay adapted here. Since the other putative substrates do not show similarity to known proteins, it will be particularly interesting to examine them further. Initial BACTH assays did not implicate proteins 00510, 02385, 00905, 17170, 01575, and 01275 as T4SS substrates. However, all of them, except 17170, do contain XVIPCD in their C-termini and, therefore, should not be eliminated as putative T4SS substrates and bactericidal effectors. Finally, since the T4SS effectors might function differently in different target bacteria, it will also be important to continue to expand the range of competing species beyond the strains of *E. coli, K. pneumoniae*, and *Pseudomonas* species used thus far (27, 28). In examining a given species, it will also be important to continue to include multiple types of strains. Indeed, in our initial study, we did not detect T4SS activity against the KPPR1 strain of *K. pneumoniae* (27), but in the present study, the T4SS and effectors TfcA, TfcB, and TfdA acted on the DMDS strain of *K. pneumoniae*. One potential direction of this expanded inquiry will be novel insights into how bacteria protect themselves against hostile T4SSs.

Aside from being in *S. maltophilia*, T4SSs are known to function in both environmental bacteria, including species of *Agrobacterium, Bradyrhizobium, Lysinibacillus, Lysobacter, Mesorhizobium, Piscirickettsia, Dinoroseobacter, Sinorhizobium, Vampirovibrio, Vibrio, Wolbachia,* and *Xanthomonas* (45, 56, 114–127), and pathogens of humans/mammals, including species of *Actinobacillus, Anaplasma*, *Bartonella*, *Bordetella, Brucella, Burkholderia, Coxiella, Ehrlichia, Escherichia, Helicobacter, Klebsiella, Legionella, Neisseria, Orientia,* and *Rickettsia* (48, 128–153). Thus, studies on the T4SS of *S. maltophilia* and its bactericidal role should have broad implications in environmental, agricultural, and medical arenas.

## MATERIALS AND METHODS

### Strains, media, and reagents

*S. maltophilia* K279a (American Type Culture Collection [ATCC] BAA-2423) was the wild-type strain used in this study and the parent for *virB10* mutant NUS15, *tfcA* mutant NUS17, *tfcB* mutant NUS19*,* and 20845 mutant NUS24 (27, 28). Other bacteria used in competition assays were *P. aeruginosa* ATCC strain 7700, *K. pneumoniae* strain DMDS, and *E. coli* strain MG1655 (27, 28, 154, 155). *E. coli* strains DH5α, BTH101, XL1-Blue, and BL21 were utilized for cloning or other assays, as indicated below. Strains were maintained on LB agar or in LB broth, with added chemicals, as detailed below. All primers used are described in Table S5A. The plasmids used are indicated below and in Table S5B.

### Two-hybrid analysis

A BACTH kit (Euromedex) was utilized to identify *S. maltophilia* proteins that bind to the T4SS coupling protein VirD4. The *virD4* gene (27) was PCR-amplified from K279a DNA (156, 157) using primers BC1 and BC2 and cloned, in-frame, into ampicillin-resistant pUT18C (65, 66). The resultant plasmid, pUT18C-VirD4, had the N-terminus of VirD4 fused with the T18 fragment of the adenylate cyclase from *B. pertussis* (65, 66). Using a series of other primer pairs (BC3–BC28), known and putative T4SS effectors were cloned, in-frame, into kanamycin-resistant pKT25 (65, 66), generating plasmids that encoded the N-termini of the effectors fused with the T25 fragment of the cyclase. In a similar way, we cloned some of the putative effector genes into the higher-copy-number pUT18C, whereas *virD4* was also cloned into lower-copy-number pKT25. Bait plasmids were co-transformed with prey plasmids into *E. coli* BTH101, a strain lacking endogenous adenylate cyclase but encoding a β-galactosidase gene (*lacZ*) under the control of a cAMP-dependent promoter (65, 66). Bacteria taken from eight colonies from each co-transformation were separately inoculated into LB broth containing 100 µg/mL carbenicillin, 50 µg/mL kanamycin, and 0.5 mM IPTG (to induce expression of the bait and prey fusions) in microtiter-plate wells. After ~20 h of incubation at 30°C, the cultures were diluted 1:5 in M63 buffer (65, 66). To lyse the bacteria and start the enzymatic reaction, “master mix” (158–160) was added, and the incubation continued at 30°C. The absorbance at 420 nm (*A*_420_) was recorded every 5 min for 20 min, and β-galactosidase activity expressed as Miller units was calculated after 20 min as before (65, 66). The BACTH assay was also utilized to assess the interaction between wild-type and mutant forms of effector 20845 and the cognate immunity protein 20840. Primers BC29 and BC30 were utilized to generate bait plasmid pUT18C-20840 that encoded a fusion of 20840 with the T18 fragment of adenylate cyclase. For use as the prey plasmids in this set of assays, we used both pKT25-20845 encoding wild-type 20845 fused with the T25 fragment of the adenylate cyclase and pKT25-H10A20845 and pKT25-H11A20845 that had similar fusions of the H10A and H11A mutant forms of 20845, respectively. To make pKT25-H10A20845 and pKT25-H11A20845, primers BC9 and BC10 were used to amplify H10A20845 from pBAD18-H10A20845 (below) and H11A20845 from pBAD18-H11A20845 (below).

### Assays for interbacterial competition

*S. maltophilia* strains competed against other bacteria that encoded *lacZ* (i.e., *E. coli* MG1655 and *K. pneumoniae* DMDS), and lysis of the targets was detected by monitoring the release of β-galactosidase, as previously described (56, 57, 77–81, 161). Target bacteria were inoculated into LB broth containing 0.2 mM IPTG (to induce *lacZ* expression), and in parallel, wild-type and mutant *S. maltophilia* were inoculated into LB broth. Following incubation at 37°C to a similar stage of growth (OD_600_ of 0.8–1.0), *S. maltophilia* strains were combined with the target bacteria at a 1:1 ratio (1–2 × 10^8^ CFU/mL each) and added to wells of a microtiter plate that had LB agar containing 0.2 mM IPTG and 40 µg/mL of CPRG, a cell-impermeable and chromogenic substrate of β-galactosidase. As controls, the target strains were mixed with LB broth. Upon incubation at 30°C, absorbance at 572 nm (*A*_572_) was recorded every 10 min for 4 h. Since *S. maltophilia* does not encode β-galactosidase (29), increases in absorbance were due to β-galactosidase released from target bacteria. The *A*_572_ values for the samples that did not contain *S. maltophilia* were subtracted from the *A*_572_ values obtained from the bacterial mixtures to account for background lysis of target cells. Finally, the absorbance at *t* = 0 was subtracted from every subsequent value, i.e., the *A*_572_ values shown represent the change in *A*_572_ over time. As an additional method for detecting antibacterial activity, the numbers of *S. maltophilia* CFU and target bacteria CFU were determined by plating aliquots from the inoculated wells at *t* = 0 h and *t* = 4 h. To enumerate the numbers of bacteria, we plated onto LB agar containing 5 µg/mL norfloxacin for *S. maltophilia* and LB agar containing 1 mM IPTG and 20 µg/mL 5-bromo-4-chloro-3-indolyl-beta-D-galacto-pyranoside (X-gal) for target bacteria. For competition against *P. aeruginosa* 7700*,* which lacks *lacZ*, bacterial mixtures were prepared as indicated above. Aliquots were added onto 0.2 µm nitrocellulose discs that had been placed on LB agar. After 4 h at 30°C, the disc was added into sterile ddH_2_O and vortexed to resuspend the entire bacterial mixture. Ten-fold serial dilutions of the cell suspension were then plated on either LB agar containing norfloxacin for *S. maltophilia* or on Vogel-Bonner agar (69) to obtain CFU for *P. aeruginosa*.

### Assay for the bactericidal activity of cloned effector 20845

We assayed for the ability of the 20845 effector to promote the killing of *E. coli*, analogously to what we did before to characterize TfcA and TfcB and what others did to study T6SS effectors (28, 76, 162). However, wild-type and mutant (H10A and H11A) forms of 20845 were tested for toxicity in *E. coli* that also carried the gene for the cognate immunity protein 20840. Thus, a DNA fragment encoding wild-type 20845 was PCR ampliﬁed using the primer pair BC31 and BC32 and cloned into ampicillin-resistant pBAD18 (163) under the control of an arabinose-inducible promoter (pBAD18-20845). To obtain the H10A form of 20845, primers BC33 and BC32 were used in PCR, and the resultant DNA fragment was cloned into pBAD18 (pBAD18-H10A20845). Given the sequence within BC33, this manipulation changed nucleotides 28–30 in the 20845 ORF to GCG, thereby replacing the histidine in the tenth position with an alanine. To make the H11A form of 20845, we used primers BC34 and BC32 in a similar way, thereby changing nucleotides 31–33 to GCG and exchanging the protein’s histidine in the eleventh position for alanine. The resultant DNA fragment was then cloned into pBAD18 (pBAD18-H11A20845). Using primers BC35 and BC36, a DNA fragment containing the 20840 gene was PCR-amplified and cloned into gentamicin-resistant pBBR1MCS-5 (164) placing the *S. maltophilia* gene under the control of an IPTG-inducible promoter (pBBR-20840). To perform the toxicity assay, pBAD18-20845, pBAD18-H10A20845, and pBAD18-H11A20845 were separately co-transformed along with pBBR-20840 into *E. coli* DH5α. As controls, vectors pBAD18 and pBBR1MCS-5 were co-transformed into DH5α as were pBAD18 and pBBR-20840. Following growth overnight at 37°C in LB broth containing 100 µg/mL carbenicillin, 20 µg/mL gentamicin, and 1% glucose (to repress expression of 20845 from the pBAD promoter), each clone was resuspended to an OD_600_ of 0.1 in LB broth containing glucose and antibiotics and incubated at 37°C with shaking. At 1 h, the cultures were centrifuged, and the bacterial pellets were washed twice with PBS to remove the glucose and resuspended in carbenicillin- and gentamicin-containing LB broth that also had either 0.4% arabinose (for induction of 20845) or 0.4% arabinose and 0.5 mM IPTG (for induction of 20845 and 20840). Bacterial survival and growth were determined by measuring the OD_600_ every hour for 6 h and by ascertaining CFU by plating 10-fold serial dilutions on LB agar containing glucose and antibiotics at 6 h.

### Assays for DNase activity of the cloned effector 20845

After overnight incubation as above, *E. coli* strain DH5α containing pBBR-20840 and either pBAD18-20845, pBAD18-H10A20845, and pBAD18-H11A20845 were inoculated into LB broth containing 1% glucose and antibiotics to an OD_600_ of 0.1 and incubated with shaking for 5 h at 37°C. At the 2-h time point, arabinose was added at 0.2% to induce expression of 20845. At the 5-h time point, the cultures were centrifuged, and the resultant pellets were resuspended to an OD_600_ of 1.0 in 5 mL of PBS. As one means of measuring DNase activity, we monitored plasmid degradation within the bacterial cells, analogously to past studies that defined T6SS DNases (82, 89–92). Thus, plasmid DNA was extracted from 2 mL of each sample using a plasmid-extraction kit (Qiagen), and the concentration of DNA in the samples was measured by Nanodrop (ThermoScientific). Two hundred ng of DNA were electrophoresed through agarose, and the separated DNAs were stained with ethidium bromide. The stained gel was imaged using the Axygen Gel Documentation System (Corning). As a second approach, we stained the bacteria with DAPI and used flow cytometry to measure the extent of DNA degradation within the cell population, as has been done to study various T6SS nucleases (67, 89). Thus, 1 mL of the above-described bacterial suspensions was stained by adding 3 µL of DAPI solution (Fisher Scientific) and incubated at room temperature for 1 h. After being diluted 1:100 in PBS, each sample was aliquoted into flow cytometry tubes and processed using the BD FACSymphony A1 cell analyzer (BD Life Sciences). In each trial, 10^5^ events were recorded for each sample where the voltage was set to 550 and 300 for forward scatter and side scatter, respectively. DAPI staining was detected using the Brilliant Violet 421 laser (450/50). Unstained and stained control samples were used to gate for DAPI staining, and data were analyzed using FlowJo software (BD Life Sciences).

### Cloning, expression, purification, and crystallization of TfcB

The *tfcB* gene (28) was amplified by PCR from the DNA of *S. maltophilia* K279a (GenBank: CAQ46442; codons 1–334) and cloned using ligation-independent cloning as described previously (165, 166) into pMCSG53 (167), which expresses protein with an N-terminal 6xHis tag followed by a tobacco etch virus (TEV) protease cleavage site and encodes ampicillin resistance and rare codons. The resulting plasmid was transformed into *E. coli* BL21(DE3) (Magic) cells (168). The protein was expressed in 3 L of High Yield M9 SeMet media (Medicilon Inc.) supplemented with 200 µg/mL ampicillin and 50 µg/mL kanamycin inoculated with an overnight starting culture (1:100 dilution) and incubated at a temperature of 37°C and rotation at 220 rpm. Protein expression was induced at OD_600_ = 1.8–2.0 by the addition of 0.5 mM IPTG, and the culture was further incubated at 25°C, with rotation at 200 rpm for 14 h (169). The cells were harvested by centrifugation at 6,117 × *g* for 10 min and then resuspended in 5 mL of lysis buffer (50 mM Tris [pH 8.3], 0.5 M NaCl, 10% glycerol, 0.1% IGEPAL CA-630) per 1 g of cell pellet. The resuspended pellet was frozen at −30°C until purification. Frozen pellets were thawed and sonicated at 50% amplitude, in 5 s (on) × 10 s (off) cycles for 20 min on ice. The lysate was cleared by centrifugation at 18,000 × *g* for 40 min at 4°C, the supernatant was collected, and the protein was purified using an ÅKTAxpress system (GE Healthcare) as before with some modifications (170). Briefly, the supernatant was loaded onto a HisTrap FF (Ni-NTA) column in loading buffer [10 mM Tris-HCl (pH 8.3), 500 mM NaCl, 1 mM Tris (2-carboxyethyl) phosphine (TCEP), and 5% glycerol]. The column was washed with 10 column volumes (cv) of loading buffer and 10 cv of high salt washing buffer (10 mM Tris-HCl [pH 8.3], 1M NaCl, 25 mM imidazole, 5% glycerol). The protein was eluted with elution buffer (10 mM Tris [pH 8.3], 500 mM NaCl, 1 M imidazole) and directly loaded onto a Superdex 200 26/600 column and separated in the loading buffer. The protein was collected and analyzed by sodium dodecyl sulfate-polyacrylamide gel electrophoresis (SDS-PAGE). The 6×His-tag was cleaved by TEV protease (1:20 protease:protein) for 18 h at room temperature during dialysis into the loading buffer. As the protein precipitated during concentration, the protein was diluted with loading buffer, resuspended, and filtered across a 0.45-µm filter. The cleaved protein was separated from recombinant TEV protease, uncleaved protein, and 6×His-tag peptide by Ni-NTA-affinity chromatography using loading buffer followed by loading buffer with 25 mM imidazole. The cleaved protein was collected in the flow through in loading buffer, analyzed by SDS-PAGE for purity and 6×His-tag cleavage, and then concentrated to 10.2 mg/mL. The purified protein was diluted to 8.4 mg/mL in 10 mM Tris-HCl (pH 8.3), 150 mM NaCl, and 1 mM TCEP and then set up for crystallization as 2 µL sitting drops (1 µL protein: 1 µL reservoir solution) in 96-well crystallization plates (Corning) using commercial Classics II, PEG’s II and Anions (QIAGEN) crystallization screens. Diffraction quality crystal of the protein that grew from the condition with 0.2 M ammonium sulfate, 0.1 M trisodium citrate (pH 5.6), 25% (wt/vol) polyethylene glycol 4000 (PEG’s II, #66) was flash cooled in liquid nitrogen for data collection.

### Structure determination of TfcB

Data sets were collected at beamline 21-ID-D of the Life Sciences-Collaborative Access Team (LS-CAT) at the Advanced Photon Source, Argonne National Laboratory. Images were indexed, integrated, and scaled using HKL-3000–2.3.15 (171). Data quality and structure refinement statistics appear in Table S4. The structure was determined by the single anomalous dispersion (SAD) method using the anomalous signal from Se-Met protein derivatives. Initial models were built using the HKL-3000 structure solution package and went through several rounds of refinement in REFMAC-5.8.0267 (172) and manual model corrections in Coot (0.9.4.1) (173). The water molecules were generated using ARP/wARP −8.0 (174) followed by additional rounds of refinement in REFMAC-5.8.0267. Structures were further refined with the Translation–Libration–Screw group corrections, which were created by the TLS Motion Determination server (175, 176). The quality control of the models during refinement and for the final validation of the structures was done using MolProbity (177) (http://molprobity.biochem.duke.edu/). Structures were deposited to the RCSB PDB (https://www.rcsb.org/) with the assigned PDB code 7r6s.

### Software for TfcB analysis and other *in silico* analyses

Docking of four molecules of GlcNAc to TfcB was done using default parameters with HADDOCK2.2 (https://wenmr.science.uu.nl/haddock2.4/) (98, 178), EDock (https://zhanggroup.org/EDock/) (100), and the HDock server (http://hdock.phys.hust.edu.cn/) (99). PyMol V2.5 software (PyMOL Molecular Graphics System, Version 2.0 Schrödinger) was used to visualize structures. The PDBsum server (https://www.ebi.ac.uk/thornton-srv/databases/pdbsum/) was used to analyze the interaction of GlcNc with TfcB and generate LigPlots. The CASTp server (179) was used to measure the volume of the groove for the chitinases. A predicted structure for 20845 was generated using AlphaFold v2.1.0 (88) and rendered using ChimeraX (180). BLASTP at the NCBI was used to identify proteins that were related to 20845. Finally, to identify alignments between the GHH-containing region of 20845 and previously identified GHH nucleases of bacteria, 12 reference sequences containing Tox-GHH2 domains were extracted from the NCBI conserved domain database. Partial alignment of these domain sequences and GHH nucleases from *P. mirabilis* and *P. aeruginosa* (85, 87), with the 20845 sequence was done with Jalview (181) using ClustalOmega (182) with default parameters.

### Statistical procedures

In all experiments, each sample or condition was assessed using at least three technical replicates, and the resultant values obtained were presented as the means and standard deviations. *P* values were determined by the two-tailed Student’s *t*-test, unless otherwise stated in a figure legend. Repeat experiments (biological replicates) were routinely performed for confirmation, as noted in the figure legends.

## Data Availability

Structures were deposited to the RCSB PDB (https://www.rcsb.org/) with the assigned PDB code 7r6s.

## AFTER EPUB

[This article was published on 4 June 2024 with the incorrect supplemental file. The supplement was corrected in the current version, posted on 1 July 2024.]

## SUPPLEMENTAL MATERIAL

Supplemental data mbio.01198-24-s0001.pdf Figures S1-S5 and Tables S1-S5.

ASM does not own the copyrights to Supplemental Material that may be linked to, or accessed through, an article. The authors have granted ASM a non-exclusive, world-wide license to publish the Supplemental Material files. Please contact the corresponding author directly for reuse.
