# Supplementary material for: Bactericidal effectors of the Stenotrophomonas maltophilia type IV secretion system: functional definition of the nuclease TfdA and structural determination of TfcB
Source: mBio. 2024 Jun 4;15(7):e01198-24. doi: 10.1128/mbio.01198-24 (PMC11253643; doi:10.1128/mbio.01198-24)
Supplement: Supplemental data — Figures S1-S5 and Tables S1-S5. [file mbio.01198-24-s0001.pdf]

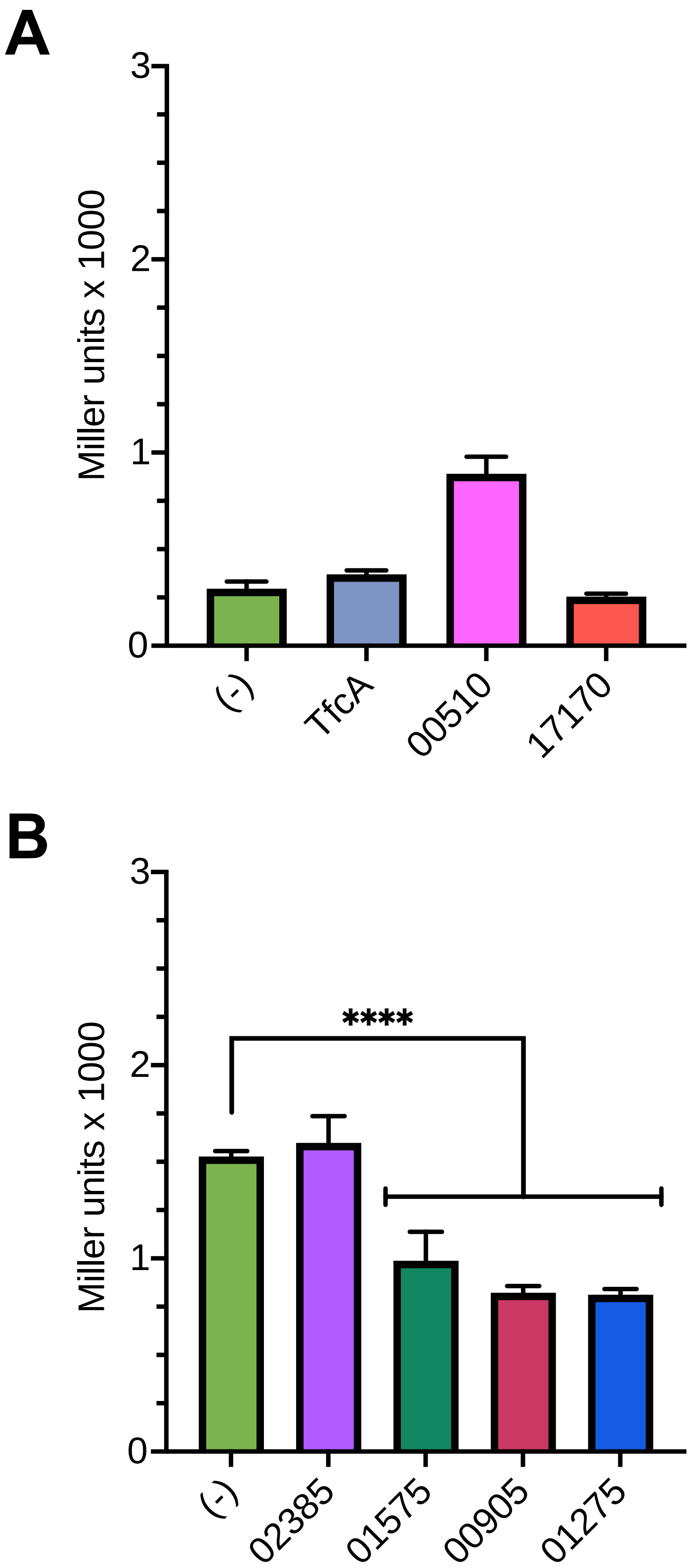

FIG S1. Further BACTH assessment of *S. maltophilia* T4SS substrate interactions. *E. coli* BTH101 containing both a "bait" plasmid expressing *S. maltophilia* VirD4 fused to the T25 fragment of adenylate cyclase (pKT25-virD4) and a "prey" plasmid derived from pUT18C that expresses the T18 fragment of the adenylate cyclase fused to either nothing (-) or the *S. maltophilia* protein TfcA, 00510, or 17170 (A) or the *S. maltophilia* protein 02385, 01575, 00905, or 01275 (B) were grown in LB broth (n = 8) with shaking for approx. 20 h at 30°C, and levels of β-galactosidase activity (Miller units) determined. Reconstituted adenylate cyclase activity was not evident for any of these clones, since there were no significant increases in β-galactosidase activity over that of the negative (-) control and in some instances, there was a slight decrease (****, $P < 0.0001$). Data are presented as means and standard deviations, and the results are representative of at least two independent experiments.

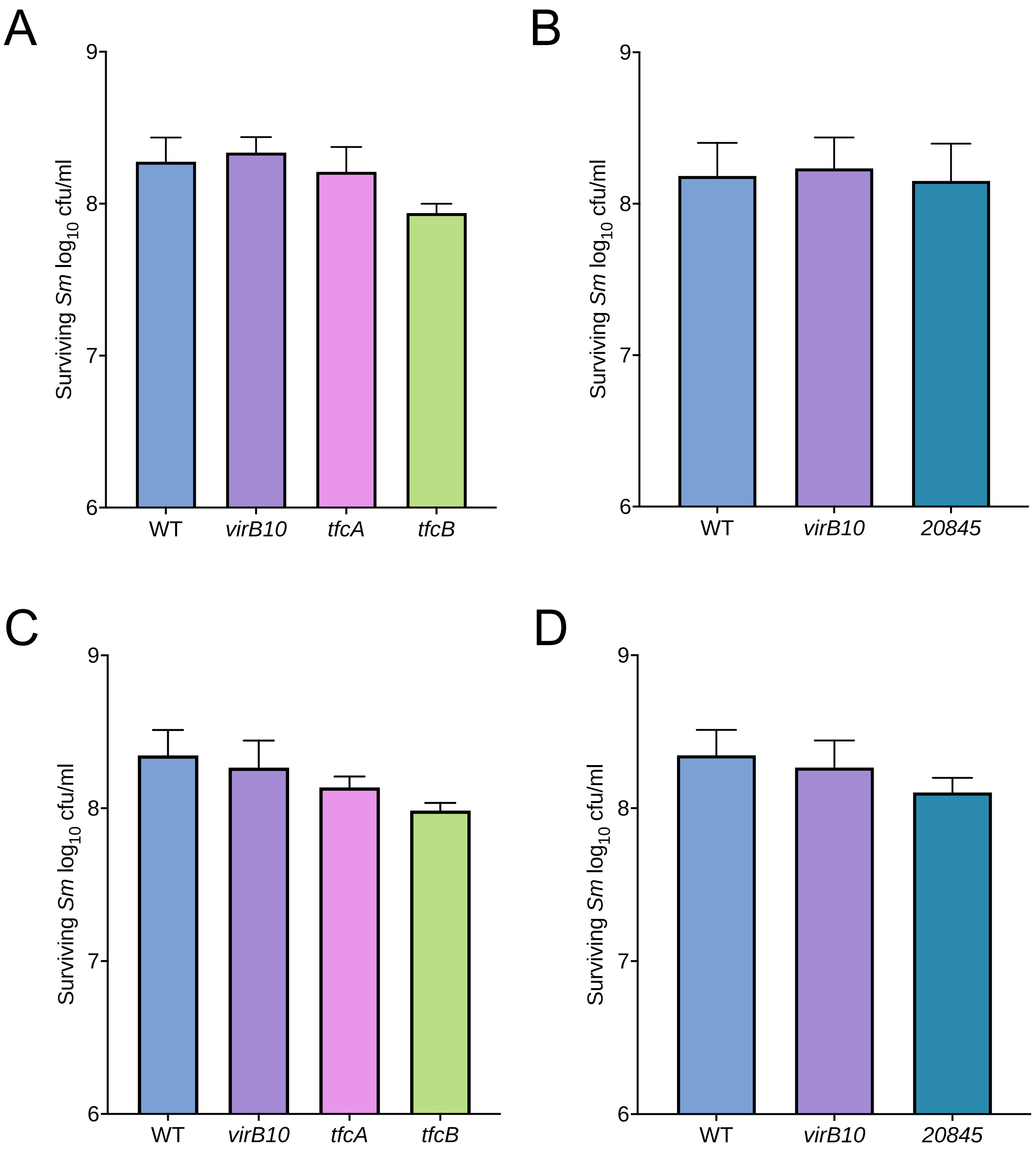

FIG S2. Numbers of *S. maltophilia* wild-type and T4SS mutants following co-incubation with strains of *E. coli* and *K. pneumoniae.* (A – D) 1-2 x 10^8 CFU/ml of *S. maltophilia* K279a (WT), *virB10* mutant NUS15 (*virB10*), *tfcA* mutant NUS17 (*tfcA*), *tfcB* mutant NUS19 (*tfcB*), and 20845 mutant NUS24 (*20845*) were mixed (*n* = 6) in a 1:1 ratio with either *E. coli* MG1655 (A, B) or *K. pneumoniae* DMDS (C, D). Following 4 h incubation at 30ºC, the numbers of remaining *S. maltophilia* (*Sm*) CFU in each of the co-cultures were determined. Data are presented as the means and standard deviations of results pooled from at least three independent experiments. There were no significant differences in the survival of the different *S. maltophilia* strains.

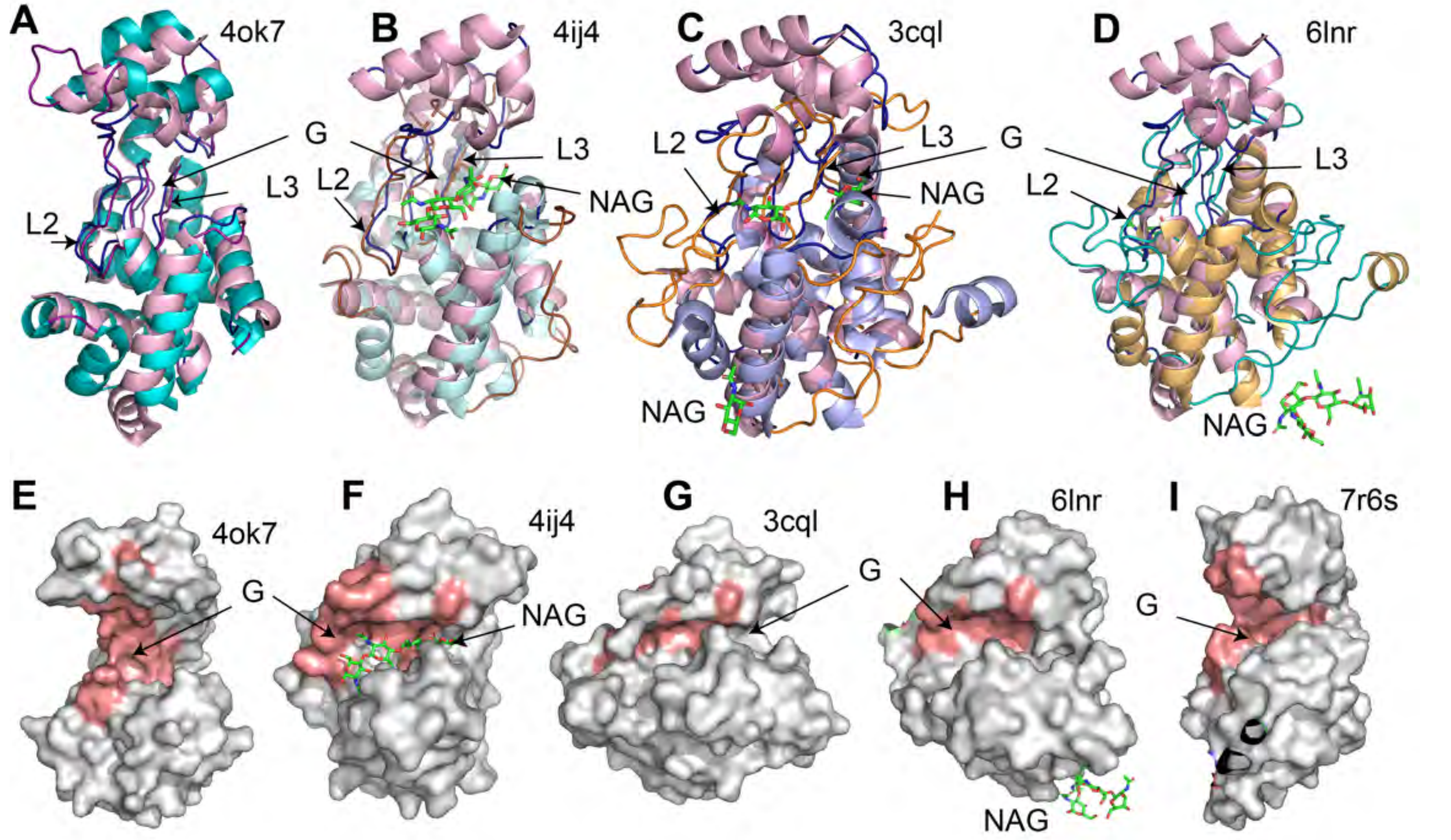

FIG S3. Structural overlay of the N-terminal domain of TfcB with other GH family 19 chitinases bound to $(GlcNAc)_4$. (A – D) Structural alignment of TfcB (pink) with its structural fold homologs (A) bacteriophage SPN1S endolysin (4ok7, light blue), (B) *B. coronatum* chitinase (4ij4, cyan), (C) *C. papaya* chitinase (3cql, purple), and (D) *S. glauca* chitinase (6lnr, wheat). Loops L2 and L3 are also indicated. The chitinases from *B. coronatum*, *C. papaya*, and *S. glauca* are shown in complex with substrate $(GlcNAc)_4$ (NAG, green) bound to the groove region (G). In conjunction to the common groove binding region, chitinases from *C. papaya* and *S. glauca* also exhibit other additional substrate binding region. (E - I) Surface representation of bacteriophage SPN1S endolysin (4ok7, E), *B. coronatum* chitinase (4ij4, F), *C. papaya* chitinase (3cql, G), *S. glauca* chitinase (6lnr, H), and TfcB (7r6s, I) showing the groove region (G) and residues from L2 and L3 in magenta.

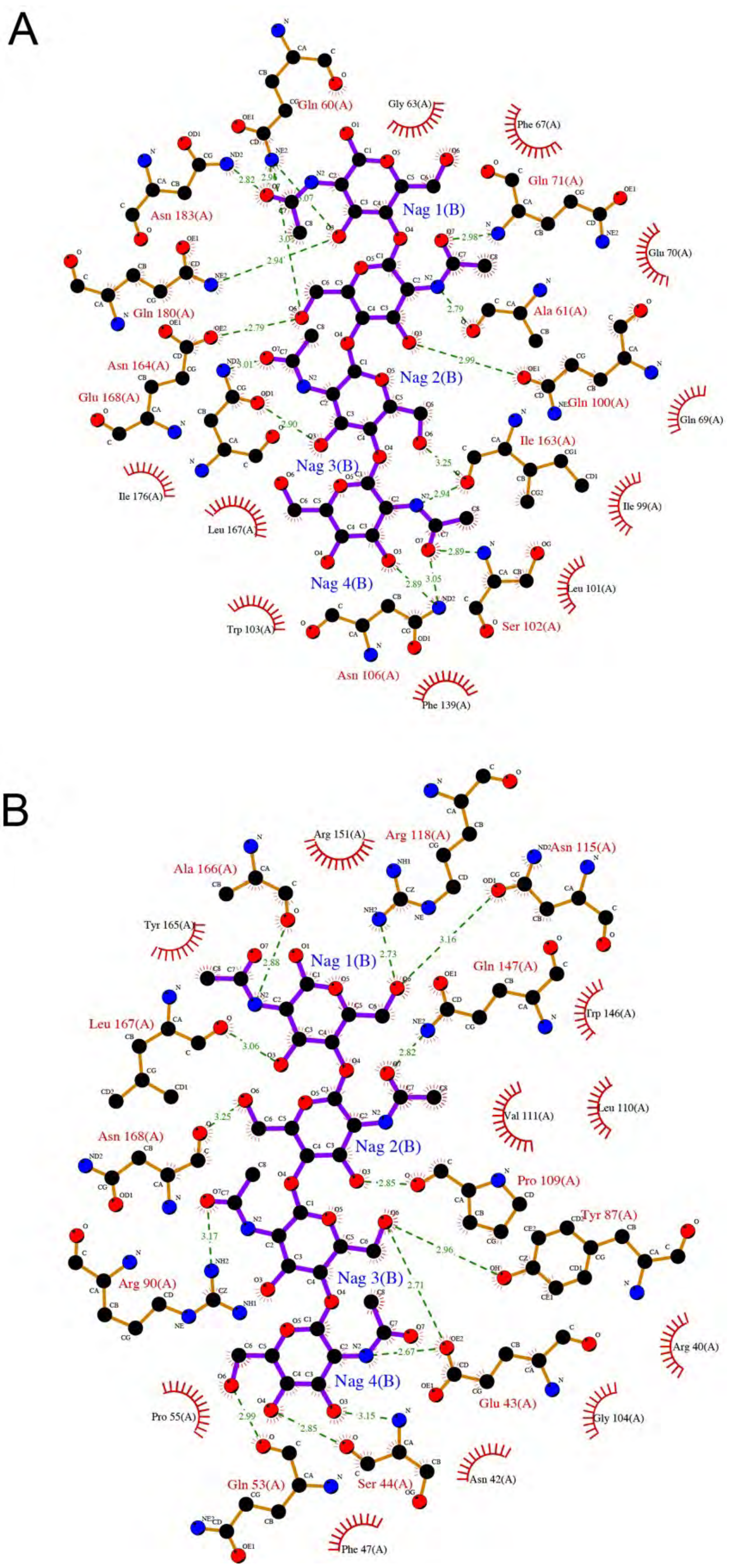

FIG S4. Ligplot depicting interaction of $(GlcNAc)_4$ with chitinase from (A) *B. coronatum* (pdb 4ij4) and (B) TfcB (pdb 7r6s). For TfcB, docking of $(GlcNAc)_4$ was done using Haddock server.

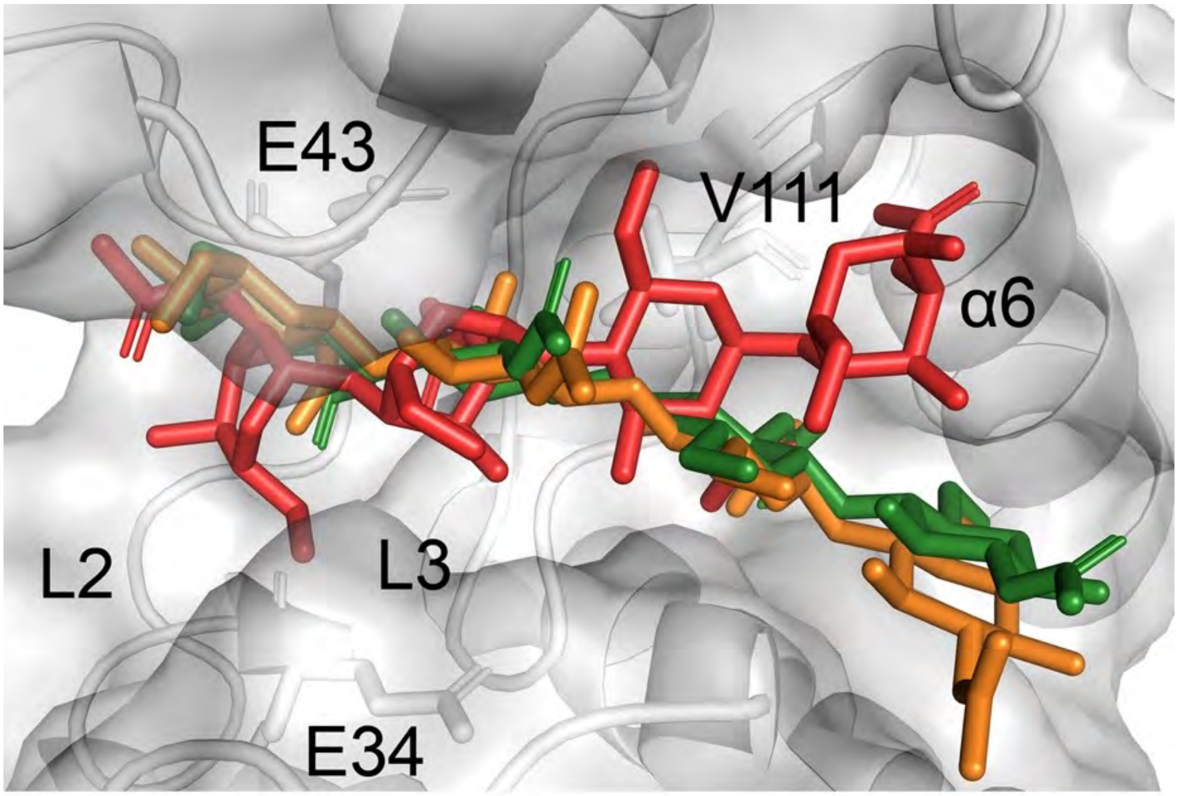

FIG S5. Docking of $(GlcNAc)_4$ to TfcB (pdb 7r6s) using server Haddock [$(GlcNAc)_4$, orange], H-Dock [$(GlcNAc)_4$, green] and E-Dock [$(GlcNAc)_4$, red].

Table S1. Presence of 20845 homologs in *Stenotrophomonas maltophilia* strains

Accession Number	Strain	Presence	E value	% Identify	% Coverage
GCF_000072485.1	K279a	yes	0	100	100
GCF_014156975.1	WP1-W18-CRE-01	yes	0	100	100
GCF_900475685.1	NCTC10498	yes	0	99.89	100
GCF_020080085.1	XL133	yes	0	99.89	100
GCF_030867045.1	11066	yes	0	99.11	100
GCF_025426215.1	CW002SM	yes	0	99.11	100
GCF_008693985.1	FDAARGOS_649	yes	0	99.11	100
GCF_027594805.1	454	yes	0	99	100
GCF_021117195.1	2013-SM15	yes	0	99	100
GCF_020641455.2	ACYCa.2H	yes	0	99	100
GCF_002208885.2	FDAARGOS_325	yes	0	99	100
GCF_011386925.1	NCTC10498	yes	0	99	100
GCF_009676425.1	sm454	yes	0	99	100
GCF_002951115.1	FDAARGOS_92	yes	0	98.89	100
GCF_023702495.1	142	yes	0	98.78	100
GCF_009676605.1	ICU331	yes	0	98.67	100
GCF_033100275.1	503	yes	0	98.55	100
GCF_020641355.2	ACYCe.8N	yes	0	98.55	100
GCF_030315995.1	a7	yes	0	98.44	100
GCF_028891445.1	HT2	yes	0	98.44	100
GCF_009676405.1	Sm53	yes	0	98.44	100
GCF_012647025.1	NEB515	yes	0	98.22	100
GCF_021117175.1	2013-SM24	yes	0	98.11	100
GCF_020641075.2	ACYCa.1J	yes	0	98.11	100
GCF_026428295.1	CYZ	yes	0	98.11	100
GCF_900475405.1	NCTC10258	yes	0	97.55	100
GCF_003812985.1	FDAARGOS_507	yes	0	97.44	100
GCF_022014735.1	PSKL2	yes	0	97.33	100
GCF_012971765.1	CF13	yes	0	96.89	100
GCF_032401205.1	SI1	yes	0	95.11	100
GCF_020641395.2	ACYCa.6E	yes	0	94.44	100
GCF_000020665.1	R551-3	yes	0	92.23	100
GCF_009676445.1	sm-RA9	yes	0	90.89	100
GCF_009618035.1	KMM 349	yes	0	90.22	100
GCF_021117255.1	2013-SM4	yes	2E-75	40.69	67
GCF_006974125.1	X28	yes	4E-75	40.09	62
GCF_016659185.1	DHHJ	yes	6E-74	39.66	61
GCF_900636655.1	NCTC13014	yes	1E-67	30.24	85
GCF_009676525.1	PEG-42	yes	5E-67	30.09	85
GCF_021441865.1	WGB211	yes	6E-67	29.97	78
GCF_003006435.1	SJTH1	yes	3E-59	28.31	85
GCF_009676565.1	PEG-173	yes	2E-58	28.22	85
GCF_020641085.2	ACYCd.9D	yes	4E-55	27.79	85
GCF_007833655.1	SM 866	yes	2E-54	27.83	85
GCF_003030985.1	W18	yes	2E-47	30.77	48
GCF_002847385.1	CSM2	yes	2E-39	30.09	51
GCF_000223885.1	JV3	yes	2E-30	30.37	52
GCF_009676465.1	SKK 55.00	no			
GCF_016126935.1	FDAARGOS_1044	no			
GCF_023277525.1	FZD2	no			
GCF_023518235.1	GYH	no			
GCF_034424605.1	HAMBI_2659	no			
GCF_029457435.1	LH-B2	no			
GCF_900186865.1	NCTC10257	no			
GCF_002138415.1	OUC_Est10	no			
GCF_009676585.1	PEG-141	no			
GCF_024734725.1	SCAID WND1-2022 (370)	no			
GCF_025642255.1	SG.Y2	no			
GCA_014171555.1	SoD9b	no			
GCF_033095765.1	STEN00241	no			
GCF_014076535.1	T50-20	no			

Table S2. Presence of 20845 related proteins in *Stenotrophomonas* species

Accession Number	Species	Strain	Presence	E value	% Identity	% Coverage
GCF_000072485.1	*S. maltophilia*	K279a	yes	0	100	100
GCF_004551575.1	*S. indicatrix*	DAIF1	yes	2E-91	35.98	74
GCF_032054965.1	*S. chelatiphaga*	CTOTU49193	yes	1E-87	40.59	70
GCF_001431485.1	*S. ginsengisoli*	DSM 24757	yes	5E-82	33.86	75
GCF_034508895.1	*S. geniculata*	FLMAT1	yes	2E-75	40.09	61
GCF_014596295.1	*S. lacuserhaii*	K32	yes	2E-66	28.50	95
GCF_014836545.1	*S. pennii*	Sa5BUN4	yes	3E-63	28.27	95
GCF_030070085.1	*S. sepilia*	ZH16	yes	1E-56	28.13	85
GCF_001431505.1	*S. daejeonensis*	JCM 16244	yes	5E-44	34.63	42
GCF_900188015.1	*S. lactitubi*	YR347	yes	3E-31	28.66	47
GCF_001704155.1	*S. rhizophila*	QL-P4	yes	5E-27	27.52	48
GCF_008710035.1	*S. cyclobalanopsidis*	TPQG1-4	no			
GCF_001431525.1	*S. koreensis*	DSM 17805	no			
GCF_019704495.1	*S. pavanii*	Y	no			
GCF_001431585.1	*S. pictorum*	JCM 9942	no			
GCF_014109845.1	*S. acidaminiphila*	T0-18	no			
GCF_013185915.1	*S. bentonitica*	DSM 103927	no			
GCF_001431415.1	*S. humi*	DSM 18929	no			
GCF_023556335.1	*S. mori*	CPCC 101365	no			
GCF_034506535.1	*S. muris*	CCV155	no			
GCF_009467805.1	*S. nematodicola*	CPCC 101271	no			
GCF_017304535.1	*S. nitritireducens*	SCN18_13_7_16_R1_B_68_91	no			
GCF_003046775.1	*S. panacihumi*	GSS15	no			
GCF_001431465.1	*S. terrae*	DSM 18941	no			
GCF_014117215.1	*S. tumulicola*	JCM 30961	no			

Table S3. Presence of 20845 related proteins in species outside of the *Stenotrophomonas* genus*

NCBI Annotation	Species	Coverage**	E value	% Identity	Accession
***AHH domain-containing protein	*Pseudomonas cichorii*	100%	0	98.5	MCV4211071.1
***uncharacterised protein	*Acinetobacter baumannii*	100%	0	97.42	SSM86812.1
hypothetical protein	*Klebsiella pneumoniae*	88%	0	97.21	HBZ8061392.1
hypothetical protein CAC00_26835	*Raoultella ornithinolytica*	88%	0	96.78	OWY84922.1
***hypothetical protein H2204_008170	*Knufia peltigerae*	100%	0	83.62	KAJ9631443.1
XVIPCD domain-containing protein	*Xanthomonas sacchari*	81%	5.00E-176	65.14	WP_244150023.1
XVIPCD domain-containing protein	*Xanthomonas nasturtii*	88%	5.00E-162	50.97	WP_064629533.1
XVIPCD domain-containing protein	*Xanthomonas arboricola*	88%	1.00E-161	50.58	WP_104607571.1
hypothetical protein FPL03_20485	*Xanthomonas citri pv. glycines*	88%	8.00E-160	50.39	QDS14102.1
hypothetical protein	*Xanthomonas campestris pv. trichodesmae*	88%	2.00E-159	50.58	MBZ3920323.1
XVIPCD domain-containing protein	*Xanthomonas citri*	88%	6.00E-159	50.49	WP_228909010.1
hypothetical protein NY67_02855	*Xanthomonas citri pv. fuscans*	88%	3.00E-158	50.58	KGP30893.1
***XVIPCD domain-containing protein	*Xanthomonas hortorum*	99%	4.00E-158	50.58	WP_176340188.1
***XVIPCD domain-containing protein	*Xanthomonas dyei*	99%	9.00E-158	50.39	WP_316690082.1
hypothetical protein GW16_00640	*Xanthomonas arboricola pv. celebensis*	88%	3.00E-157	49.61	KER88697.1
XVIPCD domain-containing protein	*Xanthomonas vasicola*	88%	6.00E-157	49.61	WP_017115093.1
XVIPCD domain-containing protein	*Xanthomonas prunicola*	88%	6.00E-157	50.19	WP_260812171.1
***XVIPCD domain-containing protein	*Xanthomonas bonasiae*	99%	6.00E-157	50.87	WP_206229743.1
***XVIPCD domain-containing protein	*Xanthomonas campestris*	99%	1.00E-156	50.58	WP_076039912.1
hypothetical protein NC00_03145	*Xanthomonas cannabis pv. phaseoli*	88%	2.00E-156	50	KGK59332.1
XVIPCD domain-containing protein	*Xanthomonas campestris pv. campestris*	88%	2.00E-156	49.22	MEB1609678.1
hypothetical protein NB99_05845	*Xanthomonas citri pv. fuscans*	87%	1.00E-155	50.4	KGK66979.1
hypothetical protein AKJ12_16110	*Xanthomonas arboricola pv. juglandis*	88%	1.00E-155	48.64	AKU51158.1
XVIPCD domain-containing protein	*Xanthomonas vesicatoria*	88%	4.00E-155	50.88	WP_229000616.1
hypothetical protein NDY24_11310	*Xanthomonas hortorum pv. pelargonii*	88%	2.00E-154	48.84	UUF00191.1
***XVIPCD domain-containing protein	*Xanthomonas cannabis*	99%	4.00E-153	50	WP_184369011.1
***XVIPCD domain-containing protein	*Xanthomonas euvesicatoria*	99%	2.00E-151	50.19	WP_280304726.1
***hypothetical protein LMG31884_01450	*Xanthomonas hydrangeae*	99%	2.00E-151	49.03	CAD7712527.1
hypothetical protein	*Xanthomonas hortorum pv. vitians*	70%	2.00E-118	47.87	NMI20179.1
hypothetical protein DB828_15670	*Xanthomonas perforans*	61%	5.00E-108	53.75	TQV22425.1
hypothetical protein	*Enterobacter hormaechei*	28%	1.00E-101	99.33	WP_223862881.1
***AHH domain-containing protein	*Xanthomonas cassavae*	70%	2.00E-100	55.02	WP_228325826.1
hypothetical protein GCM10023307_04270	*Lysobacter hankyongensis*	53%	2.00E-83	52.86	GAA4782823.1
***AHH domain-containing protein	*Xanthomonas axonopodis pv. begoniae*	41%	3.00E-54	58.49	MBO9741528.1
hypothetical protein EBB59_13250	*Lysobacter pythonis*	39%	3.00E-51	54.33	RMH87299.1
hypothetical protein	*Xanthomonas surreyensis*	90%	3.00E-51	33.33	WP_191824292.1
hypothetical protein	*Xanthomonas campestris pv. phormiicola*	90%	1.00E-50	32.94	MCC4598067.1
hypothetical protein	*Xanthomonas translucens*	91%	7.00E-50	35.06	MDR6673850.1
hypothetical protein	*Xanthomonas citri pv. Aurantifolii*	28%	6.00E-43	56.08	EFF49592.1
hypothetical protein	*Xanthomonas campestris pv. zinniae*	28%	7.00E-42	52.7	MCC4610024.1
hypothetical protein A6R71_10420	*Xanthomonas translucens pv. arrhenatheri*	26%	6.00E-39	56.83	OAX64563.1
AHH domain-containing protein	*Xanthomonas melonis*	40%	9.00E-39	56.08	WP_230434839.1
XVIPCD domain-containing protein	*Lysobacter enzymogenes*	88%	8.00E-38	32.59	WP_207522540.1
XVIPCD domain-containing protein	*Xanthomonas pisi*	24%	1.00E-35	55.91	WP_245879377.1
hypothetical protein	*Lysobacter gummosus*	89%	1.00E-34	31.85	WP_057942792.1
hypothetical protein	*Staphylococcus aureus*	12%	1.00E-30	100	MDG6745654.1
***hypothetical protein E8M68_14950	*Neisseria gonorrhoeae*	11%	1.00E-29	100	TJW98740.1
***AHH domain-containing protein	*Klebsiella pneumoniae*	11%	2.00E-29	100	HBZ8062019.1
XVIPCD domain-containing protein	*Lysobacter capsici*	91%	3.00E-26	29.64	WP_052756282.1
XVIPCD domain-containing protein	*Xanthomonas prunicola*	16%	9.00E-23	55.17	WP_260815471.1
hypothetical protein GCM10009429_09120	*Dyella marensis*	35%	1.00E-22	39.79	GAA0686448.1
hypothetical protein CFBP7900_02160	*Xanthomonas hortorum pv. carotae*	17%	4.00E-22	52.17	CAD0303474.1
zeta toxin family protein	*Lysobacter antibioticus*	39%	1.00E-18	36.15	ALN81295.1
zeta toxin family protein	*Lysobacter pythonis*	39%	5.00E-18	31.63	WP_122102183.1
putative peptidoglycan binding domain protein	*Lysobacter antibioticus*	53%	9.00E-18	28.52	ALN63048.1
***AHH domain-containing protein	*Xanthomonas vesicatoria*	12%	1.00E-17	66.15	WP_229000451.1
***AHH domain-containing protein	*Xanthomonas hortorum*	12%	1.00E-17	63.08	WP_192824388.1
***AHH domain-containing protein	*Xanthomonas campestris*	12%	4.00E-17	67.69	WP_116887281.1
***AHH domain-containing protein	*Xanthomonas hortorum*	12%	4.00E-17	67.69	WP_233397413.1
***AHH domain-containing protein	*Xanthomonas citri*	12%	6.00E-17	63.08	WP_228909012.1
*AHH domain-containing protein	*Xanthomonas arboricola*	12%	2.00E-16	67.69	WP_181141488.1
hypothetical protein	*Xanthomonas fragariae*	17%	4.00E-16	50	WP_002809613.1
XVIPCD domain-containing protein	*Lysobacter antibioticus*	31%	4.00E-16	39.29	WP_152566071.1
***AHH domain-containing protein	*Xanthomonas vasicola*	12%	5.00E-16	63.08	WP_017119589.1
XVIPCD domain-containing protein	*Lysobacter capsici*	26%	7.00E-16	42.75	WP_057920373.1
zeta toxin family protein	*Luteibacter aegosomaticola*	26%	8.00E-16	32.37	WP_247329904.1
hypothetical protein	*Xanthomonas sacchari*	12%	9.00E-16	66.67	WP_319019792.1
XVIPCD domain-containing protein	*Vulcaniibacterium gelatinicum*	24%	1.00E-15	44.19	WP_147652458.1
XVIPCD domain-containing protein	*Lysobacter maris*	36%	1.00E-15	32.49	WP_145985488.1
XVIPCD domain-containing protein	*Thermomonas fusca*	32%	1.00E-15	36.05	WP_138346833.1

* Partial listing, as we have included only one representative strain per species / pv.

** Full length (white), nuclease domain (purple), nuclease + central region (yellow), central (green), central + XVIPCD (pink), XVIPCD (blue)

*** Contains a GHH- not AHH- nuclease motif

Table S4. Data quality and refinement statistics for the TfcB structure*

**PDB Accession Code**	**7r6s**
**Data Collection**	
Space group	$P2_1$
Unit cell parameters (Å; º)	*a* = 52.44, *b* = 90.67, *c* = 72.07;
	α = 90.00, β = 103.99, γ = 90.00
Resolution range (Å)	30.00 – 1.90 (1.93 - 1.90)
No. of reflections	51,491 (2,592)
R_{merge} (%)	8.2 (92.8)
Completeness (%)	98.9 (99.6)
$\langle I/\sigma(I)\rangle$	20.1 (2.0)
Multiplicity	5.9 (5.6)
Wilson *B* factor	34.6
**Refinement**	
Resolution range (Å)	28.71 - 1.90 (1.95 - 1.90)
Completeness (%)	98.9 (99.3)
No. of reflections	48,349 (3,736)
R_{work}/R_{free}, (%)	18.8/23.1 (31.7/33.9)
Protein chains/atoms	2/4,875
Ligand/Solvent atoms	30/306
Mean temperature factor (Å^2)	46.0
**Coordinate Deviations**	
R.m.s.d. bonds (Å)	0.004
R.m.s.d. angles (º)	1.269
**Ramachandran plot**	
Favored (%)	99.0
Allowed (%)	1.0
Outside allowed (%)	0.0

* Values in parentheses are for the outer shell

Table S5A. Primers and restriction enzyme (RE) sites used in this study

Name	RE	5' to 3' Sequence	Gene
BC1	XbaI	GCGGCGTCTAGAGGTGTTGCCCAGTAAGAAGTAT	RS14330
BC2	KpnI	GAAGGGTACCTGATTAGACGTTAAGCATTGGAAC	RS14330
BC3	XbaI	GCGGCGTCTAGAGATGAGACCTCTAACCAATGCA	RS19100
BC4	KpnI	GAAGGGTACCTGACTAGCCCATGCGCATCGCTGT	RS19100
BC5	XbaI	GCGGCGTCTAGAGatgAGCGGACTTACCGATAG	RS02375
BC6	KpnI	GCGGCGGGTACCtcaGCCCATGCGCATGGCGT	RS02375
BC7	XbaI	GCGGCGTCTAGAGATGGCCGAGATCACCCCCAAC	RS02400
BC8	KpnI	GCGGCGGGTACCTTACCCCAACCGAGGCGCAGG	RS02400
BC9	XbaI	GCGGCGTCTAGAGATGGCCGACACACCGGTTTTCC	RS20845
BC10	EcoRI	GCGGCGGAATTCTCAACCCAACCGCGGCCCCT	RS20845
BC11	XbaI	GCGGCGTCTAGAGGTGTCGACCGATAGAGAGTCGCA	RS14255
BC12	KpnI	GCGGCGGGTACCCTACAGGGATCGAGAACGCTGCT	RS14255
BC13	XbaI	GCGGCGTCTAGAGATGAGTGGATTGACCGAGCGA	RS14405
BC14	KpnI	GAAGGGTACCTGATCATCCCATCCGCATCGCATG	RS14405
BC15	XbaI	GCGGCGTCTAGAGATGCCGGAGATGGATATCAGCG	RS00510
BC16	EcoRI	GCGGCGGAATTCTCAACGTCCTCCCATCGCG	RS00510
BC17	XbaI	GCGGCGTCTAGAGATGGAGCAGGCAACACGCTACACC	RS02385
BC18	KpnI	GCGGCGGGTACCTTACTGCTCCTTCAACCGCAGCG	RS02385
BC19	XbaI	GCGGCGTCTAGAGATGGCACAGCATGACTACAGC	RS00905
BC20	KpnI	GAAGGGTACCTGATTAGACGCCCATCGCCTTCTG	RS00905
BC21	XbaI	GCGGCGTCTAGAGGTGACCATCACCTCTCAGGACTA	RS14245
BC22	KpnI	GCGGCGGGTACCTCAGGCCTGCAGGCTGCGGGTCT	RS14245
BC23	XbaI	GCGGCGTCTAGAGATGGCCACCATGAACATTTCG	RS17170
BC24	KpnI	GAAGGGTACCTGATCACTTGCGGTTCCGTGCACGCTCA	RS17170
BC25	XbaI	GCGGCGTCTAGAGATGGAGCGTACTGAAGACGCTGG	RS01575
BC26	KpnI	GCGGCGGGTACCTTACCTTGAGAACGATAGCCCGTCC	RS01575
BC27	XbaI	GCGGCGTCTAGAGATGCCAACCAACATCGATACCC	RS01275
BC28	EcoRI	GCGGCGGAATTCTTACGCCATCATGGCCCGCGG	RS01275
BC29	XbaI	GCGGCGTCTAGAGATGATGGAAGCCCCCGTGACC	RS20840
BC30	BamHI	GCGGCGGGATCCCTAGAGATCGGCTACATCGCG	RS20840
BC31	EcoRI	GCGGCGGAATTCGAATGGCCGACACACCGGTTTTC	RS20845
BC32	XbaI	GCGGCGTCTAGATCAACCCAACCGCGGCCCCTG	RS20845
BC33	EcoRI	GCGGCGGAATTCGAATGGCCGACACACCGGTTTTCCAGGGAGCGCATCTGATCGAA	RS20845
BC34	EcoRI	GCGGCGGAATTCGAATGGCCGACACACCGGTTTTCCAGGGACACGCGCTGATCGAA	RS20845
BC35	BamHI	GAAGGGATCCATGATGGAAGCCCCCGTGACC	RS20840
BC36	XbaI	GCGGCGTCTAGACTAGAGATCGGCTACATCGCG	RS20840

Table S5B. Plasmids made for this study

Plasmid	Descrption
pUT18C-virD4	*virD4* cloned into BACTH pUT18C (high copy) plasmid
pKT25-19100	*19100* cloned into BACTH pKT25 (low copy) plasmid
pKT25-02375	*02375* cloned into BACTH pKT25 (low copy) plasmid
pKT25-02400	*02400* cloned into BACTH pKT25 (low copy) plasmid
pKT25-20845	*20845* cloned into BACTH pKT25 (low copy) plasmid
pKT25-tfcB	*tfcB* cloned into BACTH pKT25 (low copy) plasmid
pKT25-14405	*14405* cloned into BACTH pKT25 (low copy) plasmid
pKT25-00510	*00510* cloned into BACTH pKT25 (low copy) plasmid
pKT25-02385	*02385* cloned into BACTH pKT25 (low copy) plasmid
pKT25-00905	*00905* cloned into BACTH pKT25 (low copy) plasmid
pKT25-tfca	*tcfA* cloned into BACTH pKT25 (low copy) plasmid
pKT25-17170	*17170* cloned into BACTH pKT25 (low copy) plasmid
pKT25-01275	*01275* cloned into BACTH pKT25 (low copy) plasmid
pUT18C-20840	*20840* cloned into BACTH pKT25 (low copy) plasmid
pKT25-H10A20845	H10A form of *20845* cloned into BACTH pKT25 (low copy) plasmid
pKT25-H11A20845	H11A form of *20845* cloned into BACTH pKT25 (low copy) plasmid
pKT25-virD4	*virD4* cloned into BACTH pKT25 (low copy) plasmid
pUT18C-00510	*00510* cloned into BACTH pUT18C (high copy) plasmid
pUT18C-17170	*17170* cloned into BACTH pUT18C (high copy) plasmid
pUT18C-02385	*02385* cloned into BACTH pUT18C (high copy) plasmid
pUT18C-01575	*01575* cloned into BACTH pUT18C (high copy) plasmid
pUT18C-00905	*00905* cloned into BACTH pUT18C (high copy) plasmid
pUT18C-01275	*01275* cloned into BACTH pUT18C (high copy) plasmid
pBAD18-20845	WT form of *20845* cloned into pBAD18
pBAD18-H10A20845	H10A form of *20845* cloned into pBAD18
pBAD18-H11A20845	H11A form of *20845* cloned into pBAD18
pBBR-20840	*20840* cloned into pBBR1MCS-5
